# Maternal Dietary Restriction Alters Offspring’s Sleep Homeostasis

**DOI:** 10.1371/journal.pone.0064263

**Published:** 2013-05-31

**Authors:** Noriyuki Shimizu, Sachiko Chikahisa, Yuina Nishi, Saki Harada, Yohei Iwaki, Hiroaki Fujihara, Kazuyoshi Kitaoka, Tetsuya Shiuchi, Hiroyoshi Séi

**Affiliations:** 1 Department of Integrative Physiology, Institute of Health Biosciences, The University of Tokushima Graduate School, Tokushima, Japan; 2 Student Lab, University of Tokushima Faculty of Medicine, Tokushima, Japan; Rikagaku Kenkyūsho Brain Science Institute, Japan

## Abstract

Nutritional state in the gestation period influences fetal growth and development. We hypothesized that undernutrition during gestation would affect offspring sleep architecture and/or homeostasis. Pregnant female mice were assigned to either control (fed *ad libitum*; AD) or 50% dietary restriction (DR) groups from gestation day 12 to parturition. After parturition, dams were fed AD chow. After weaning, the pups were also fed AD into adulthood. At adulthood (aged 8–9 weeks), we carried out sleep recordings. Although offspring mice displayed a significantly reduced body weight at birth, their weights recovered three days after birth. Enhancement of electroencephalogram (EEG) slow wave activity (SWA) during non-rapid eye movement (NREM) sleep was observed in the DR mice over a 24-hour period without changing the diurnal pattern or amounts of wake, NREM, or rapid eye movement (REM) sleep. In addition, DR mice also displayed an enhancement of EEG-SWA rebound after a 6-hour sleep deprivation and a higher threshold for waking in the face of external stimuli. DR adult offspring mice exhibited small but significant increases in the expression of hypothalamic peroxisome proliferator-activated receptor α (*Pparα*) and brain-specific carnitine palmitoyltransferase 1 (*Cpt1c*) mRNA, two genes involved in lipid metabolism. Undernutrition during pregnancy may influence sleep homeostasis, with offspring exhibiting greater sleep pressure.

## Introduction

Timing, duration, and depth of sleep are controlled by the interaction of the time of day (circadian control) and by the duration of prior wakefulness (homeostatic control) [1], [2], [3]. Sleep homeostasis is expressed via electroencephalogram (EEG) slow wave activity (SWA) during non-rapid eye movement (NREM) sleep. SWA in NREM sleep is used as a parameter of sleep pressure, need for sleep, or sleepiness [2]. Recently SWA has been well documented to have an important role in synaptic plasticity [4], [5], [6]. It has also been demonstrated that metabolic function, including adenosine regulation, is critically involved in sleep homeostasis [7], [8], [9].

It has been reported that peroxisome proliferator-activated receptors (PPARs) and AMP-activated protein kinase (AMPK) play key roles in the regulation of sleep homeostasis [10], [11]. PPARs are transcription factors belonging to the nuclear receptor family, and are closely related to the regulation of lipid metabolism [12], [13], [14]. AMPK acts as an efficient sensor of cellular energy states regulating glucose and lipid metabolism [15], [16], [17]. AMPK activity is altered in response to the intracellular AMP/ATP ratio. The activation of PPARs by treatment with bezafibrate, a PPAR pan-agonist, augments SWA in NREM sleep [10]. In addition to PPARs, AMPK activity also changes SWA without affecting sleep duration [11]. In addition, sleep deprivation (SD) activates AMPK [11], [18], [19], while intracerebroventricular (i.c.v.) administration of 5-aminoimidazole-4-carboxamide riboside (AICAR), an activator of AMPK, augments SWA during NREM sleep, whereas compound C, an inhibitor of AMPK, suppresses SWA [11].

The nutritional state during the gestation and/or lactation period influences fetal growth and development. Low birth weight (LBW) induced by maternal undernutrition is reported to be a crucial risk factor for metabolic disease such as obesity, diabetes, or cardiovascular disease [20], [21]. Many studies have shown that not only metabolic regulation but also brain functions such as emotional behavior, locomotor activity, and learning are all highly influenced by undernutrition during pregnancy [22].

As both PPARs and AMPK are activated under fasting conditions [23], [24], [25], it is expected that their function may be associated with undernutrution during gestation and/or LBW. We then hypothesized that adult offspring mice with embryonic undernutrition would exhibit changes in sleep architecture and/or homeostasis. In this study we found that offspring mice display larger SWA during NREM sleep with normal sleep timing and duration, and an exaggerated rebound of SWA against SD.

## Materials and Methods

### Animals and Experimental Design

Experiments were performed with Jcl:ICR mice (Slc Inc., Shizuoka, Japan). Mice (aged 10–12 weeks) were maintained on a 12-hour light/dark (L/D) cycle (lights on at 0900) at a controlled ambient temperature (23±1°C). Virgin female mice were caged with male mice. Pregnancy was dated with vaginal plugs (day 0), and pregnant female mice were housed individually with *ad libitum* access to standard chow (MF, Oriental Yeast Co., LTD, Tokyo, Japan). On gestation day 12, pregnant female mice were randomly assigned to either control (fed *ad libitum*; AD) or dietary restriction (DR) groups. Food intake of DR mothers was restricted to 50% of controls from gestation days 12 to parturition. After parturition, litter size was equalized to ten in both AD and DR groups. Mothers received chow in the AD condition after parturition. Pups were weaned at 3 weeks, and fed standard chow AD afterwards up to the age of 8–9 weeks. All experiments were performed in male mice. The Animal Study Committee of Tokushima University (No. 12051) approved these experiments, and we performed them in accordance with Guidelines for the Care and Use of Animals approved by the Council of the Physiological Society of Japan.

### Sleep and Body Temperature Recordings

Adult offspring male mice were anesthetized with a cocktail of ketamine (100 mg/kg, Daiichi-Sankyo, Tokyo, Japan) and xylazine (25 mg/kg, Sigma, St. Louis, MO, USA). A telemetric device (TA10TA-F20; Data Sciences Int., USA) for recording body temperature and spontaneous activity was implanted in the peritoneal cavity. Stainless steel miniature screw electrodes (M1.7-3, Unique Medical, Tokyo, Japan) were implanted in the skull (1.0 mm anterior to the bregma, 1.0 mm right lateral to the midline; 2.5 mm posterior to the bregma, 2.5 mm left lateral to the midline) to record the EEG. Teflon-coated stainless steel wires (AS633, Cooner Wire, Chatsworth, CA, USA) were implanted in the neck muscles on both sides to record the electromyogram (EMG). After 2 weeks of recovery, the mice were transferred to plastic cages (20 cm×24 cm×30 cm) in a soundproof recording room and allowed 3 days of habituation. Thereafter, mice were connected through an electrical slip ring (T13EEG; Air Precision, Paris, France) to a polygraph (RM-6100; Nihon Kohden, Tokyo, Japan) with a computer flat cable, and then to a computer-assisted data acquisition system CED 1401 data processor (CED, Cambridge, UK). After the habituation period, polygraphic recordings of body temperature, spontaneous activity, EEG, and EMG were collected continuously, throughout the L/D cycle. Telemetric signals of body temperature were sampled by Dataquest ART (Data Sciences Int., USA). Spontaneous activity was also recorded via signal strength of the telemetric device for body temperature. EEG and EMG signals were sampled by the Spike2 acquisition program (Cambridge Electronic Design, UK). Offline sleep scoring was first done via Spike2 software, and carefully verified by visual assessment of the EEG and EMG activities. Vigilance states were classified over 6-s epochs as wakefulness, NREM, or rapid eye movement (REM) sleep. NREM sleep was characterized by a continuous, slow, high-voltage EEG and low-voltage EMG activity. REM sleep was characterized by low-voltage EEG with continuous theta waves and total suppression of the EMG. Fast Fourier Transform using the Spike2 analysis program (Cambridge Electronic Design, UK) calculated the EEG power spectrum in the epoch determined to be NREM sleep. The sampling rate for EEG/EMG data collection was 100 Hz. The EEG delta and theta frequency band was set at 0.5–4.0 Hz and 4.0–8.0 Hz, respectively. Each value of power was presented as a percentage of the total power (0.5–50 Hz). In this study, we used delta/theta value as a parameter of SWA [26]. Body temperature, spontaneous activity, sleep/wake durations, and EEG delta/theta ratio were averaged for hourly intervals. After the 24-hour baseline-recording period, 6-hour SD by gentle touching with a brush was carried out, starting at 0900 (Zeitgeber time 0; ZT 0). Immediately after the SD, the recovery period was recorded continuously for 18 hours.

In order to estimate the phenotypic sleep pressure in mice, we measured the awaking threshold against external sensory stimuli in mice. Each mouse was set for sleep recording, and then a cage of mice was put on the shaker (rocking mixer; As One, Japan). Shaking was started 30 seconds after the appearance of EEG delta wave activity. The latency from the start of shaking to awaking determined by EEG and EMG was used as a parameter of sleep pressure. The procedure of shaking and latency measurement was performed 5 times and the latency was averaged for each mouse.

### Measurement of Plasma Levels of Triglycerides, Free Fatty Acids (FFAs), Ketone Bodies, and Glucose

At the time of decapitation, trunk blood was collected for measurement of triglycerides, FFAs, and ketone body levels. Plasma triglycerides and FFAs were determined by GPO-HDAOS (triglycerides) and ACS-ACOD (FFAs) enzyme assays using an automatic biochemical analyzer system (HITACHI 7180, Hitachi, Tokyo, Japan). Ketone bodies (β-hydroxybutyrate and acetoacetate) were measured by an automatic analyzer system JCA-BM12 (JEOL, Tokyo, Japan) using reagents for measurement of ketone bodies by enzymatic assay (Kainos Laboratories, Tokyo, Japan). Glucose was detected from tail blood by a glucose biosensor (LifeScan, Inc., Milpitas, CA, USA).

### In vivo Metabolic Testing

Glucose tolerance was assessed after glucose intraperitoneal (i.p.) injection (2 g/kg for mice aged 14 weeks) in unrestrained awake mice after a 16-hour fast. Insulin tolerance tests (1 unit/kg for mice aged 14 weeks, Sigma Chemical Co., St. Louis, MO, USA) were performed in mice after a 6-hour fast (ZT6).

### Real Time RT-PCR Analysis

For molecular analyses, fetal mice were sacrificed at ZT9-10, and then liver and whole brain were extracted at gestation day 17. Adult offspring mice at the age of 8–9 weeks were sacrificed at ZT4-5, and then liver and brain were extracted. The brain was sectioned coronally on ice with a brain slicer (Muromachi Kikai, Tokyo, Japan). Coronal brain sections were divided into fractions of hypothalamus, cerebral cortex, hippocampus, and striatum by a brain matrix. Brain and liver tissue were immediately frozen in liquid nitrogen, and stored at −80°C until use. Total RNA in fetal and adult offspring mice was isolated following Takara’s RNA isolation protocol (RNAiso Plus; Takara Bio, Shiga, Japan). cDNA in fetal and adult offspring mice was generated from each RNA sample using a High-Capacity cDNA Transcription Kit (Applied Biosystems, Foster, CA, USA). We used predesigned, gene-specific TaqMan probes and primer sets to assess expression of the genes indicated in Table S1. Real-time PCR was performed with an Applied Biosystems 7900HT real-time PCR system using TaqMan Universal PCR Master Mix (Roche Applied Science, Mannheim, Germany) according to the manufacturer’s instructions. Cytoplasmic beta-actin (β-actin, encoded by *Actb*) was used for an endogenous quantitative control, and values were normalized to *β-actin* mRNA expression.

### Pharmacological Treatments and Injection Procedures

To investigate the effect of caffeine on behavior, caffeine (15 mg/kg, Sigma Chemical Co) was administered i.p. 30 min before the forced swim test. The detailed procedure of the forced swim test is described in Protocol S1. In order to evaluate the effect of caffeine on sleep, caffeine (5 mg/kg) was injected at ZT0 during sleep recordings. The caffeine dose was selected according to a previous study [27].

### Statistics

Results are expressed as means ± SEM. Changes in body weight, body temperature, spontaneous activity, sleep architecture and EEG delta/theta ratio were analyzed by repeated measures one-way or two-way analysis of variance (ANOVA) followed by Scheffe’s post-hoc test. The results of latency for awaking following cage shaking and latency to sleep following a caffeine injection were analyzed by unpaired Student’s t-test. The results of behavioral tests, real-time RT-PCR, blood glucose, and measurements of plasma substances were analyzed by Mann-Whitney U test. P<0.05 was assumed to indicate statistical significance.

## Results

### Body Weight

**Figure 1:**
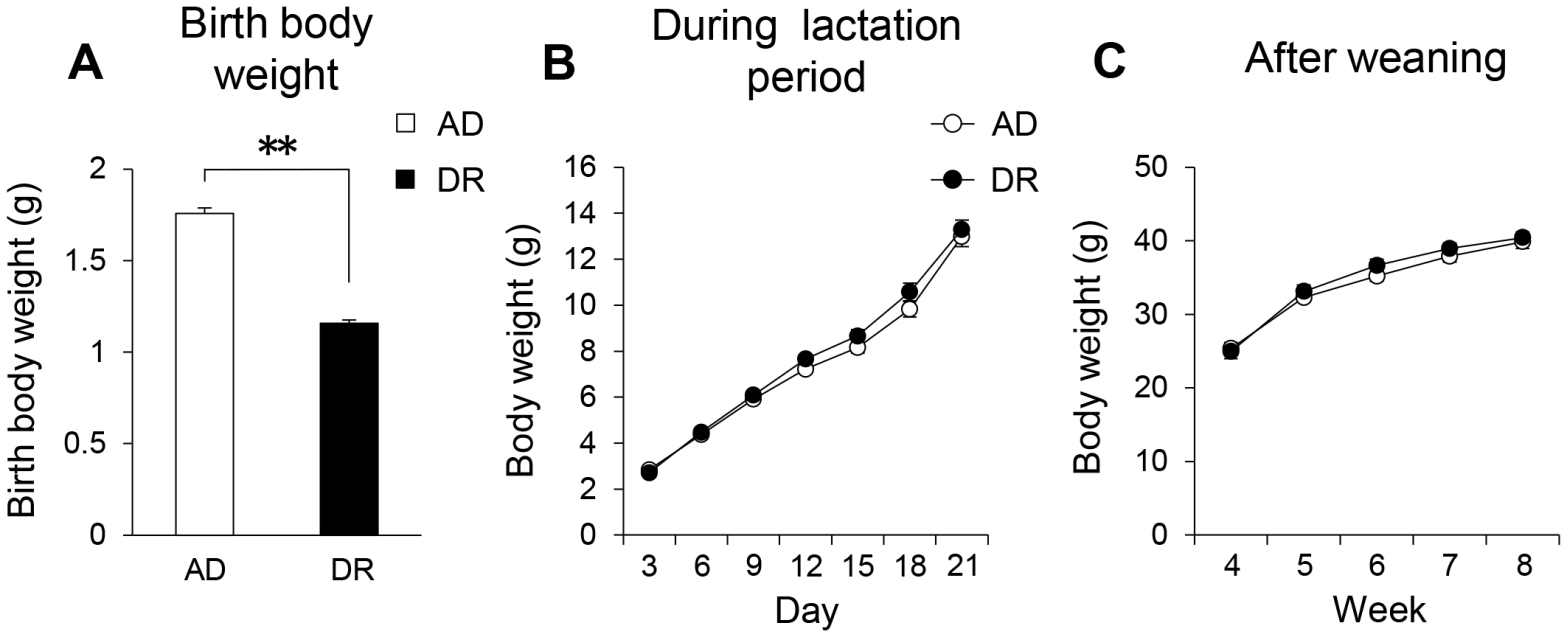
Birth body weight (A) and body weight changes during the lactation period (B) and after weaning (C) up to 8 weeks in offspring mice. Open bars and circles indicate AD mice. Closed bars and circles indicate DR mice. Data represent means ± SEM (A; n = 22–23, B; n = 36–38, C; n = 16). **p<0.01 indicates a significant difference.

The dietary restriction (DR) female mice showed significantly less body weight gain during the 4 days before parturition and just after parturition (Figure S1A). DR female mice displayed a marked decrease in blood glucose (Figure S1B). However, there were no significant differences in the number of either live births or dead births (Figure S1C, D). The ratio of males to females was not significantly different between control (fed *ad libitum*; AD) and DR offspring mice (Figure S1E). DR offspring mice exhibited significantly reduced body weights at birth (Figure 1A). However, as early as the third postnatal day, the significant differences in body weight had already disappeared (Figure 1B). That is, the DR mice exhibited LBW accompanied by an accelerated catch-up growth (CUG). Up to 8 weeks of age, when the sleep recordings and behavioral tests were carried out, no significant differences were observed in body weight between the two groups (Figure 1C).

### Sleep Architecture and Homeostasis

**Figure 2:**
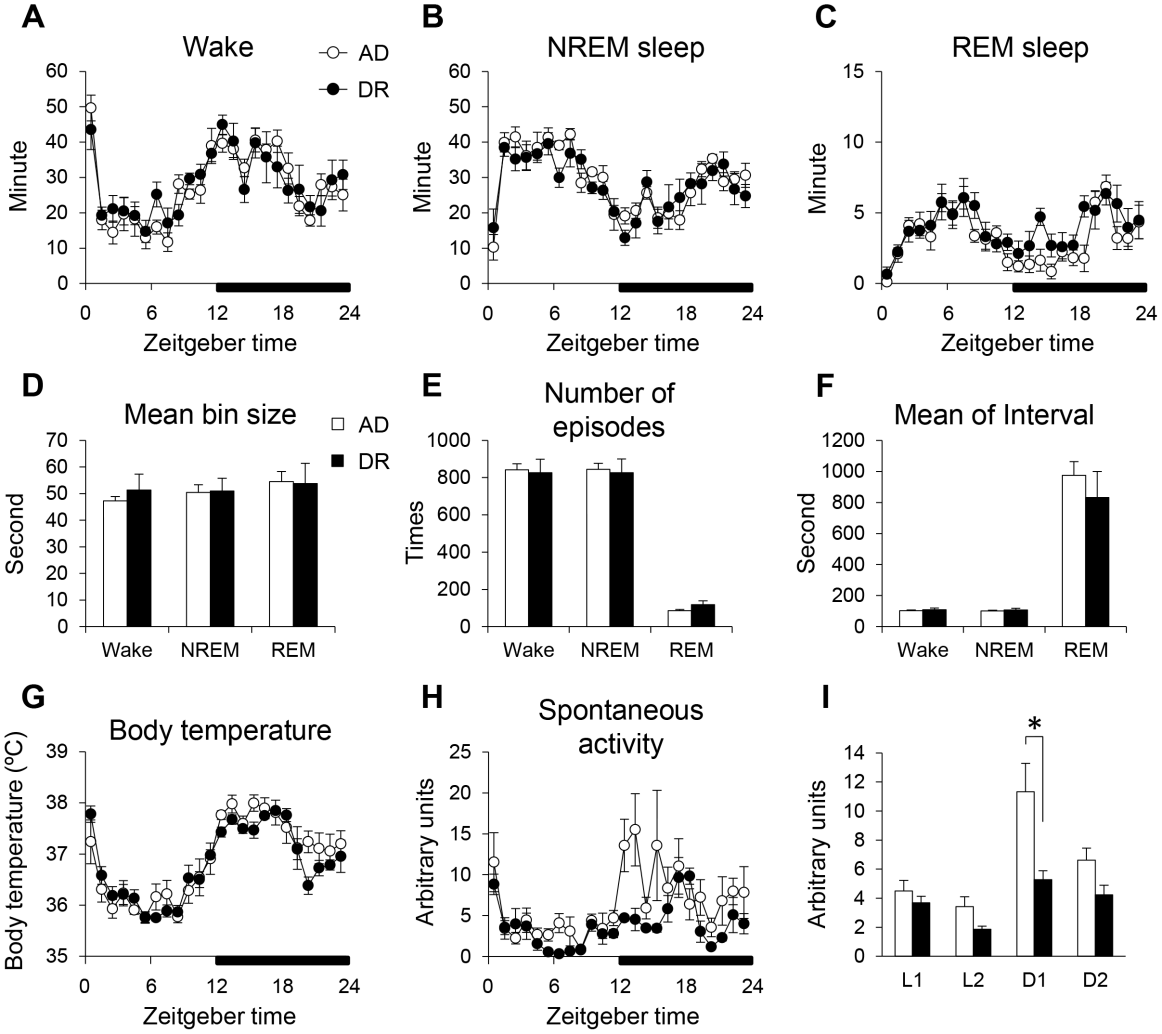
The influence of dietary restriction during gestation on sleep/wake architecture, body temperature, and spontaneous activity in adult offspring mice. Diurnal changes of wakefulness (A), NREM sleep (B), and REM sleep (C). Mean bin size (D), number of episodes (E), and mean interval (F) in each sleep/wake state for all 24-hour recordings. Hourly time course of body temperature (G) and spontaneous activity (H). Spontaneous activity was averaged for each 6-hour period (I) across ZT0-6 (L1), ZT6-12 (L2), ZT12-18 (D1), and ZT18-24 (D2). Open bars and circles indicate AD mice. Closed bars and circles indicate DR mice. Data represent means ± SEM (A–F; n = 6, G–I; n = 5). *p<0.05 indicates a significant difference.

**Figure 3:**
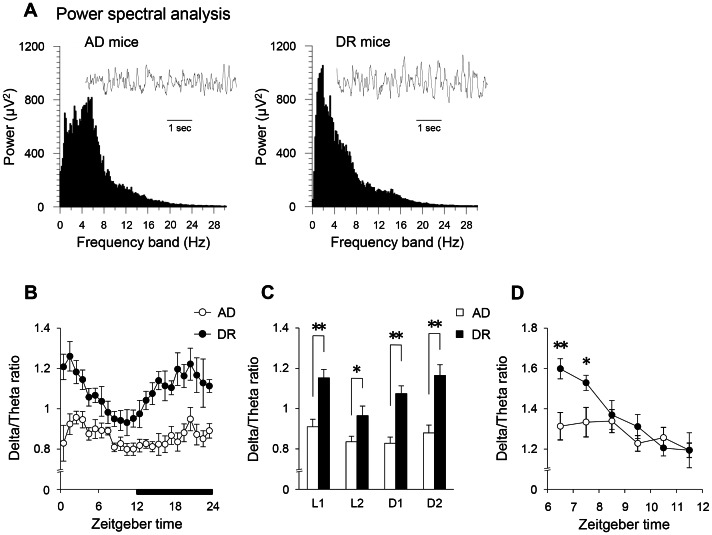
The influence of dietary restriction during gestation on sleep homeostasis in adult offspring mice. Power spectral analysis of EEG during NREM sleep (A). Hourly time course changes of EEG delta/theta ratio in NREM sleep (B), and the averages for each 6-hour period (C) across ZT0-6 (L1), ZT6-12 (L2), ZT12-18 (D1), and ZT18-24 (D2). Six-hour changes of the rebound rate of delta/theta ratios after sleep deprivation (D). Open bars and circles indicate AD mice. Closed bars and circles indicate DR mice. Data represent means ± SEM (A–D; n = 6). **p<0.01 and *p<0.05 indicate a significant difference.

**Figure 4:**
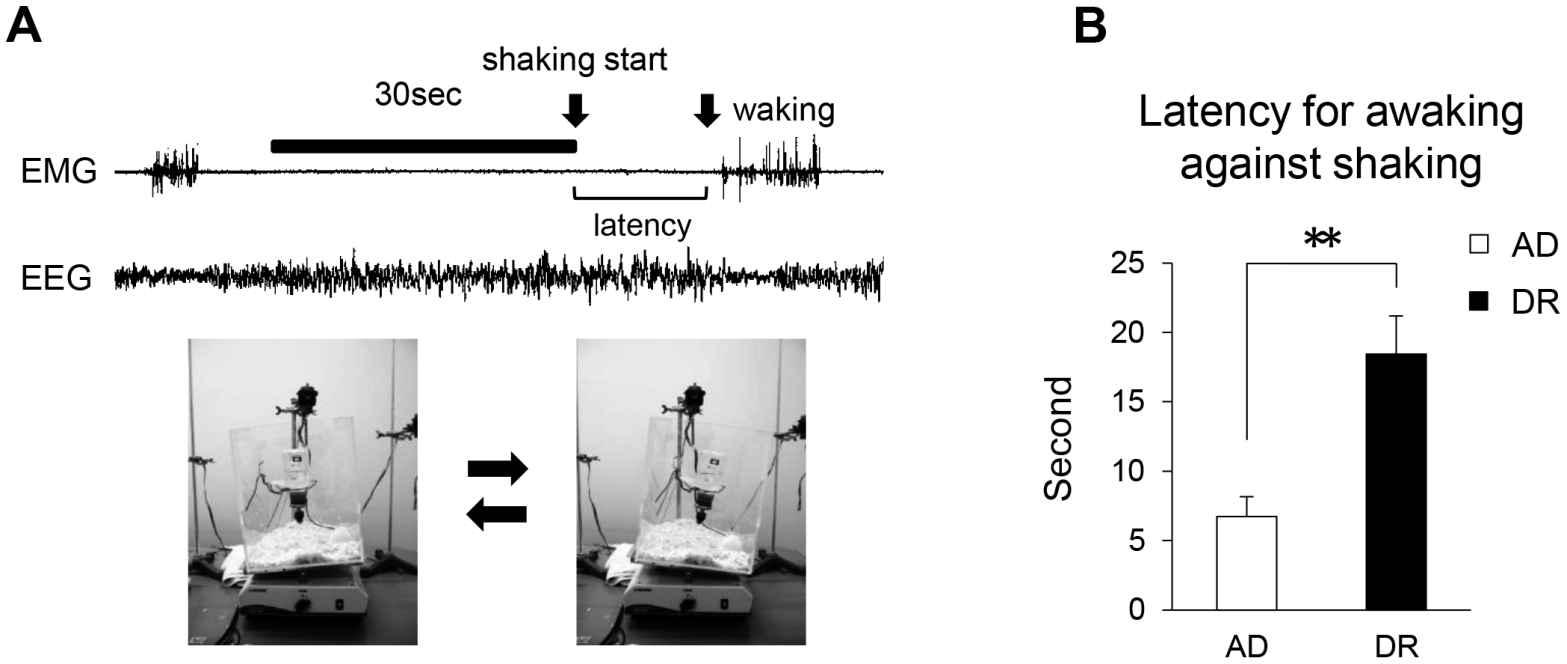
Threshold for waking by external stimuli (cage shaking) in adult offspring mice. Photos of the experimental setting for estimating waking threshold in mice against external sensory stimuli (A). Cage shaking was started 30 seconds after the continuous appearance of EEG delta waves as shown in upper traces. We measured the latency from the start of shaking to EEG arousal. The latency for waking following shaking stimuli (B). Open bars indicate AD mice. Closed bars indicate DR mice. Data represent means ± SEM (B; n = 6). **p<0.01 indicates a significant difference.

No significant changes were observed in the diurnal pattern and amount of wake, NREM, and REM sleep between the two groups (Figure 2A–C). Additionally, mean bin size, number of episodes, and mean interval of sleep/wake cycles in DR mice were also not changed (Figure 2D–F). The body temperature and its diurnal variation in DR mice were not largely modified (Figure 2G), partially and indirectly suggesting normal thermoregulation and/or circadian rhythmicity. In comparison, DR mice displayed lower spontaneous activity, especially in the first half of the dark period (Figure 2H, I). However, DR mice exhibited increased power in the delta frequency band (Figure 3A) and a significant enhancement in their EEG delta/theta ratio (SWA) in NREM sleep compared with AD mice over 24 hours (Figure 3B, C). The percent power of EEG delta power in NREM sleep also significantly increased over 24 hours (Figure S2A). Furthermore, although there was large inter-individual variation, an increase was seen even in the raw delta power in DR mice (Figure S2B). In contrast, DR mice did not display changes in EEG delta/theta ratios in either wake or REM sleep. The EEG delta/theta ratio after a 6-hour SD in DR mice was significantly enhanced compared with the AD mice during the first two hours of the recovery period (Figure 3D). The latency for waking following cage shaking (Figure 4A) was significantly longer in the DR mice (Figure 4B). In addition, the latency for waking against lights-off conditions also tended to be longer in DR mice (Figure S3). These results suggest that the DR mice have a higher threshold for waking by external stimuli.

### Metabolic States

We previously found the involvement of PPARs, nuclear receptors for lipid or glucose metabolism, or AMPK, a cellular energy (ATP/AMP) sensor, in the sleep homeostasis. In order to investigate the mechanism of the change in sleep homeostasis in DR offspring mice, we therefore evaluated the metabolic condition in those mice.

**Figure 5:**
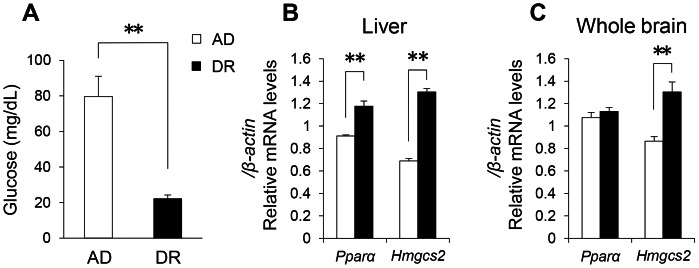
Metabolic state in fetal stage (gestation day 17). Blood glucose (A) and gene expression related to the regulation of lipid metabolism in liver (B) and whole brain (C). Open bars indicate AD mice. Closed bars indicate DR mice. Data represent means ± SEM (A–C; n = 6). **p<0.01 indicates a significant difference.

At gestation day 17, the fetuses of DR mice showed a marked decrease in blood glucose (Figure 5A). They also showed increases in the expression of *Pparα* and 3-hydroxy-3-methylglutaryl-Coenzyme A synthase 2 (HMGCS2, encoded by *Hmgcs2*) mRNAs in the liver (Figure 5B). Furthermore, the expression of brain *Hmgcs2* mRNA was also enhanced in DR mice (Figure 5C). As *Hmgcs2* is a target gene of PPARα and a key enzyme in ketogenesis [28], [29], [30], we consider that the fetuses of DR mice were under fasting conditions that produced ketogenic responses not only in the liver but also in the brain.

**Figure 6:**
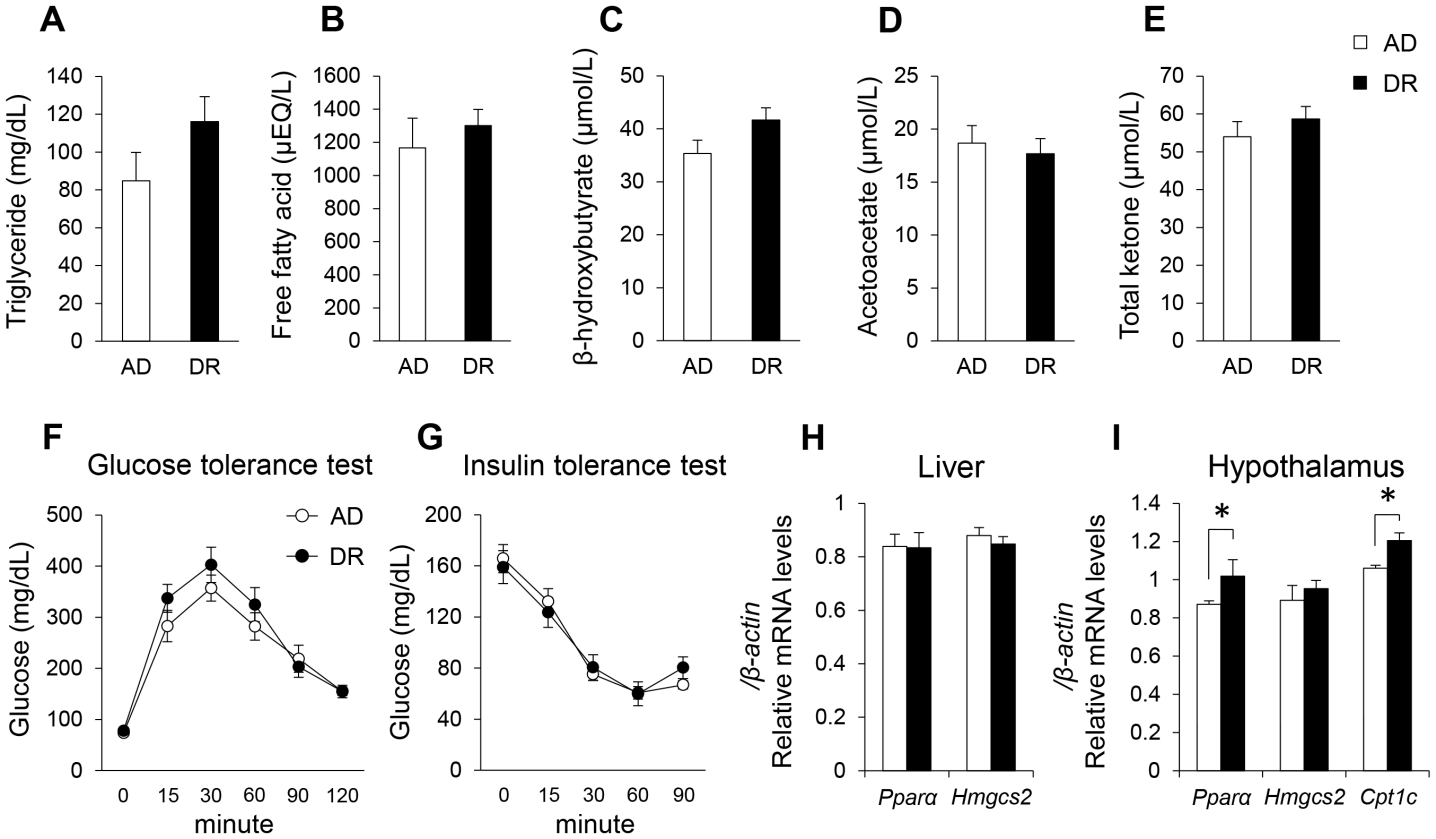
Metabolic state in adulthood (8–9 weeks). The plasma levels of triglycerides (A), free fatty acids (FFAs; B), β-hydroxybutyrate (C), acetoacetate (D), and ketone bodies (E). Glucose tolerance test (GTT; F) and insulin tolerance test (ITT; G) in adulthood. Gene expression related to the regulation of lipid metabolism in liver (H) and hypothalamus (I). Open bars and circles indicate AD mice. Closed bars and circles indicate DR mice. Data represent means ± SEM (A–E; n = 6, F, G; n = 5–6, H; n = 7, I; n = 4–6). *p<0.05 indicates a significant difference.

At the age of 8–9 weeks, no significant differences were observed in plasma levels of triglycerides, FFAs, β-hydroxybutyrate, acetoacetate, and total ketone between the two groups (Figure 6A–E). Furthermore, there were no significant differences in the glucose tolerance test (GTT) or the insulin tolerance test (ITT) between the two groups (Figure 6F, G). In the liver, no significant differences were observed in *Pparα* and *Hmgcs2* mRNAs (Figure 6H). In the hypothalamus, DR mice showed a small but significant increase in *Pparα* and brain-specific carnitine palmitoyltransferase 1 (CPT1C, encoded by *Cpt1c*) mRNA expression (Figure 6I). Regarding feeding-related neuropeptides, no significant differences were observed in the expression of neuropeptide Y (NPY, encoded by *Npy*), agouti-related protein (AGRP, encoded by *Agrp*), pro-opiomelanocortin-alpha (POMC1, encoded by *Pomc1*), and prepro-orexin (OX, encoded by *Ppox*) mRNAs in the hypothalamus (Figure S7A). Also, there were no significant differences in the mRNA expression of various glucose metabolism-related enzymes, transporters, or receptors in the hypothalamus (Figure S7B).

Protein levels of AMPKα and phosphorylated AMPKα (p-AMPKα), and their ratio (pAMPKα/AMPKα) in hypothalamus and cortex were not significantly different between the two groups (Figure S8A–H).

### Effects of Caffeine

**Figure 7:**
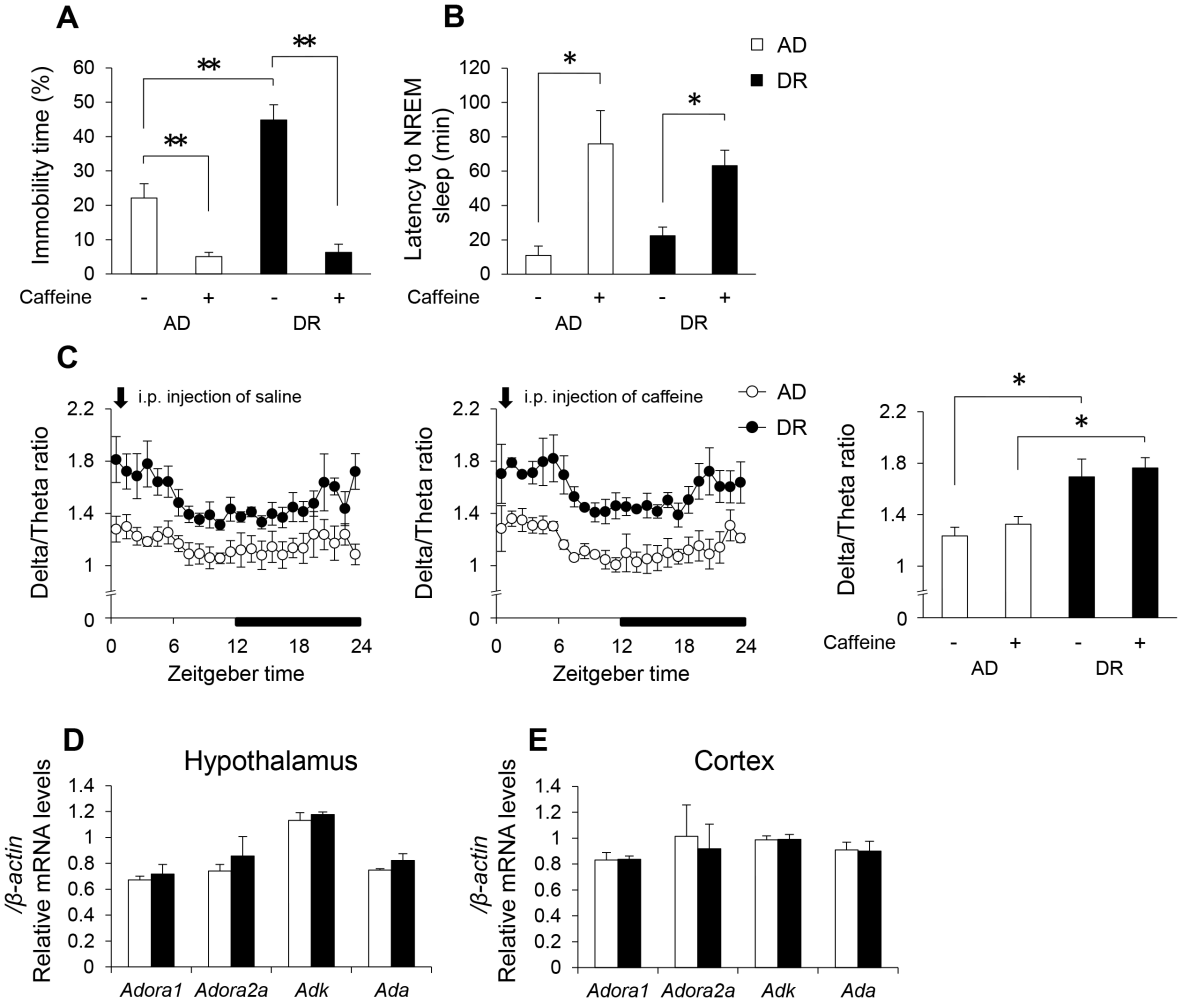
Adenosinergic system responsiveness in adult offspring mice. The influence of caffeine on immobility time (A) in the forced swim test. The effect of caffeine on latency to the onset of sleep (B) and EEG delta/theta ratios in NREM sleep (C). Gene expression related to the regulation of adenosine signaling in the hypothalamus (D) and cortex (E). Open bars and circles indicate AD mice. Closed bars and circles indicate DR mice. Arrows indicate the injection time (ZT0). Data represent means ± SEM (A; n = 9, B, C; n = 3, D, E; n = 6–7). **p<0.01 and *p<0.05 indicate a significant difference.

Caffeine is a pan-antagonist at adenosine receptors. As DR mice are considered to exhibit larger sleep pressure and adenosinergic function is well documented to be critical for sleep homeostasis [7], [8], [9], we hypothesized that adenosine or related systems would be involved in the behavioral and sleep changes in DR mice. A single i.p. injection of caffeine significantly elevated swimming behavior in DR mice in the forced swim test (Figure 7A). Although caffeine lengthened the latency to sleep in both AD and DR mice as reported in a previous study [27], differences in SWA were not observed (Figure 7B, C). There were also no significant changes in adenosine A1 receptor (ADORA1, encoded by *Adora1*), adenosine A2a receptor (ADORA2, encoded by *Adora2*), adenosine kinase (ADK, encoded by *Adk*), or adenosine deaminase (ADA, encoded by *Ada*) mRNA expression in either hypothalamus or cortex (Figure 7D, E).

## Discussion

Studies in animal models have shown that early undernutrition has a great impact on developmental programming, causing permanent changes in a variety of physiological functions including higher brain function [22]. We found in this study for the first time that sleep homeostasis is also affected by prenatal nutritional condition. Our DR adult offspring mice born with LBW showed an enhancement of SWA during NREM sleep, which is accepted to be a parameter of sleep pressure or sleep intensity. DR mice also displayed an exaggerated rebound of SWA against a 6-hour SD. Although SWA during NREM sleep is well known to be proportional to the length or activity of preceding wakefulness [1], [2], DR mice did not exhibit significant changes in the structure of their sleep-wake cycle, and their spontaneous activity was rather decreased. These data indicate that the enhanced SWA in DR mice is caused by alterations not preceding wakefulness but sleep homeostatic function itself, and that DR mice have exaggerated homeostatic sleep function. As DR mice have a higher threshold for waking by external stimuli (such as cage shaking and lights-off conditions), with normal timing and duration of sleep, we believe that DR mice actually exhibit a greater sleep pressure.

The mRNA expression of *Pparα* and *Hmgcs2*, a main target gene of PPARα [28], [29], [30], was increased in the fetal DR mice, indicating that they were under a fasting condition. In fact, we observed a marked decrease in blood glucose in the fetal DR mice. The enhancement of ketogenesis was observed in the fetal DR mice not only in the liver but also in the brain. At 8–9 weeks old, the DR adult offspring mice showed small but significant increases in the mRNA expression of *Pparα* and *Cpt1c* in the hypothalamus. *Cpt1* is another target gene of PPARα, and its activation indicates enhanced lipid β-oxidation in mitochondria [31]. It is suggested that hypothalamic PPARα function may still be activated even in the adult offspring mice. We previously found that bezafibrate, a pan-agonist of PPARs, induces increases of SWA in NREM sleep [10]. Furthermore, we also observed that 6-hour SD slightly but significantly increased mRNA expression of *Pparα* in mouse brain (Figure S9), just as seen in the DR mice. When the pressure to sleep is augmented, PPARα seems to be activated. It is hypothesized that the up-regulation of PPARα in DR offspring mice may be involved in altered sleep homeostasis.

Monoaminergic neural groups such as serotonergic, noradrenergic, and dopaminergic circuits are considered to be a major part of the waking system [3], [32], [33]. However, we did not observe differences in released transmitter or metabolite concentrations in dialysates from the hippocampus (Figure S5A–C) or striatum (Figure S5E–G) with respect to these classes. Furthermore, there were no large changes in the mRNA expression of monoamine-related receptors, transporters, or enzymes (Figure S5D, H). Therefore, monoaminergic neural function also seems to not be the cause of altered sleep homeostasis in DR mice.

Caffeine, a pan-antagonist of adenosine receptors, activated swimming behavior in DR mice. As DR mice are considered to have more sleep pressure and adenosinergic function is well documented to be critical for sleep homeostasis [7], [8], [9], we had hypothesized that adenosine or related systems would be involved in the behavioral and sleep changes in DR mice. However, we did not find significant changes in mRNA expression relating to the adenosinergic system (including *Adora1*, *Adora2*, *Adk*, or *Ada*) in DR mice. Furthermore, although the latency to sleep was lengthened by caffeine in both mice groups, SWA in NREM sleep was not affected. It is therefore considered that, although behavioral activation systems respond to antagonism of adenosine receptors, changes in sleep homeostasis caused by maternal undernutrition are independent of the adenosinergic function.

It is well known that LBW accompanied by postnatal CUG caused by maternal undernutrition is a crucial risk factor for metabolic dysfunction, including type 2 diabetes [34], [35]. Indeed, we observed a marked increase in body weight in DR adult male and female mice (Figure S10) in later stage (32 weeks). Metabolic dysfunction may influence sleep mechanisms. However, our DR mice, at least at the age of sleep recording, did not exhibit abnormal plasma triglyceride, FFA, ketone body concentrations, or impaired GTT or ITT. At this stage, DR mice do not yet seem to display pathological changes in metabolic function, and changes in sleep homeostasis do not depend on it.

DR mice exhibited a modest augmentation of anxiety- and depression-like behavior (Figure S4A, I). However, antidepressant drugs (an SSRI, an SNRI, and a dopaminergic stimulant) could not alleviate depression-like behavior in DR mice (Figure S6). Furthermore, we did not detect any changes in the brain serotonergic, noradrenergic, or dopaminergic function (Figure S5A–H). These results indicate that depression-like behavior in DR mice may not model depression seen in humans. Alternatively, the DR mice may have some impairment in terms of sensitivity to these anti-depressant drugs. Although DR mice display normal moving distance and speed over a short period in the open field test (Figure S4B, C), their activity did not increase either in the forced swim test or during the first half of the dark period. It seems that when increased activity is required, DR mice are unable to adjust their activity in such conditions. Combined with our data demonstrating enhanced sleep pressure after SD, we believe that DR mice may be vulnerable against prolonged or activated wakefulness. This fatigability of DR mice may cause the lower mobility in the forced swim test.

In this study, sleep homeostasis was shown to be significantly modified by maternal undernutrition, although underlying mechanisms remain to be further investigated. It is possible that some sleep disturbance in human adulthood may be caused by the mother’s inadequate nutritional condition during pregnancy.

## Supporting Information

Figure S1 The influence of dietary restriction during gestation on maternal body weight changes, blood glucose, and live birth. Body weight changes before and after parturition in mother mice (A). Maternal blood glucose concentration (B) on gestation day 17. Live births (C), dead births (D), and ratio of male to female live births (E). Open bars and circles indicate AD mice. Closed bars and circles indicate DR mice. Data represent means ± SEM (A; n = 6–9, B; n = 2, C, D; n = 11, E; n = 7–8). **p<0.01 and *p<0.05 indicate a significant difference.
(PPTX)

Figure S2 The influence of dietary restriction during gestation on delta power in NREM sleep (A, B) in adult offspring mice. Open circles indicate AD mice. Closed circles indicate DR mice. Data represent means ± SEM (A, B; n = 6).
(PPTX)

Figure S3 Threshold for waking by external stimuli (lights off) in adult offspring mice. The latency for awaking against lights-off conditions. Open bars indicate AD mice. Closed bars indicate DR mice. Data represent means ± SEM (n = 6).
(PPTX)

Figure S4 The influence of dietary restriction during gestation on anxiety- and depression-like behaviors in adult offspring mice. Anxiety-like behavior was assessed by open field test, light-dark transition, and elevated plus maze. Time spent in the center area (A), total distance (B), and average speed (C) were assessed in the open field test. Number of transitions (D), latency to enter the light area for the first time (E), and time spent in the light area (F) were evaluated in the light-dark transition test. On the elevated-plus maze, time spent in open arms (G) and number of entries into open arms (H) were evaluated. Depression-like behavior was assessed by the forced swim test. Immobility time (I) was evaluated. Open bars indicate AD mice. Closed bars indicate DR mice. Data represent means ± SEM (A–I; n = 14). **p<0.01 and *p<0.05 indicate a significant difference.
(PPTX)

Figure S5 Monoaminergic system responsiveness in adult offspring mice. *In vivo* microdialysis. The change in extracellular concentration of serotonin (5-HT), its metabolite (5-HIAA), and norepinephrine (NE) before and after the forced swim test (A–C) in the hippocampus. The change in extracellular concentration of dopamine (DA) and its metabolites (DOPAC, HVA) before and after the forced swim test (E–G) in the striatum. Gene expression related to the regulation of serotonin signaling (D) such as 5-hydroxytryptamine receptor 1A (HTR1A, encoded by *Htr1a*), 5-hydroxytryptamine receptor 2C (HTR2C, encoded by *Htr2c*), solute carrier family 6, member 4 (SLC6A4, encoded by *Slc6a4*), tryptophan hydroxylase 1 (TPH1, encoded by *Tph1*), tryptophan hydroxylase 2 (TPH2, encoded by *Tph2*), and monoamine oxidase A (MAOA, encoded by *Maoa*) in the hippocampus. Gene expression related to the regulation of dopamine signaling (H) such as dopamine receptor D1A (DRD1A, encoded by *Drd1a*), dopamine receptor D2 (DRD2, encoded by *Drd2*), dopamine receptor D5 (DRD5, encoded by *Drd5*), solute carrier family 6, member 3 (SLC6A3, encoded by *Slc6a3*), tyrosine hydroxylase (TH, encoded by *Th*), and catechol-O-methyltransferase (COMT, encoded by *Comt*) in the striatum. Open bars indicate AD mice. Closed bars indicate DR mice. Data represent means ± SEM (A–C; n = 4, D; n = 6, E–G; n = 4, H; n = 7).
(PPTX)

Figure S6 Total time of immobility and changes in DR offspring mice treated with vehicle (Vhe; saline) or therapeutic drugs in the forced swim test. As therapeutic drugs, we used fluoxetine (Flu), imipramine (Imi), and phenelzine (Phe). Each therapeutic drug was injected intraperitoneally 30 min before the forced swim test. Open bars indicate AD mice. Closed bars indicate DR mice. Data represent means ± SEM (n = 9–10). **p<0.01 indicates a significant difference.
(PPTX)

Figure S7 Gene expression related to the feeding regulation (A) and the regulation of glucose metabolism (B) such as hexokinase 1 (HK1, encoded by *Hk1*), hexokinase 2 (HK2, encoded by *Hk2*), phosphofructokinase, muscle (PFKM, encoded by *Pfkm*), solute carrier family 2, member 1 (SLC2A1, encoded by *Slc2a1*), solute carrier family 2, member 3 (SLC2A3, encoded by *Slc2a3*), lactate dehydrogenase A (LDHA, encoded by *Ldha*), and lactate dehydrogenase B (LDHB, encoded by *Ldhb*) in the hypothalamus. Open bars indicate AD mice. Closed bars indicate DR mice. Data represent means ± SEM (A, B; n = 6–7).
(PPTX)

Figure S8 The protein levels of AMPKα, p-AMPKα, and p-AMPKα/AMPKα ratio in the hypothalamus (A–D) and cortex (E–H). Open bars indicate AD mice. Closed bars indicate DR mice. Data represent means ± SEM (A–H; n = 5).
(PPTX)

Figure S9 The effect of 6-hour sleep deprivation on the mRNA expression of *Pparα*, *Pparβ*, and *Pparγ* in mouse brain. Open bars indicate control (Con) mice. Closed bars indicate sleep-deprived (SD) mice. Data represent means ± SEM (n = 6). *p<0.05 indicates a significant difference.
(PPTX)

Figure S10 Body weight at adulthood in offspring male (aged 32 weeks) and female (aged 28–34 weeks) mice. Open bars indicate AD mice. Closed bars indicate DR mice. Data represent means ± SEM (n = 14–26). **p<0.01 indicates a significant difference.
(PPTX)

Table S1 List of gene-specific TaqMan probes and primers used for real-time RT-PCR.
(PPTX)

Protocol S1 Supplemental Methods.
(DOC)

## References

[1] Daan S, Beersma DG, Borbely AA (1984) Timing of human sleep: recovery process gated by a circadian pacemaker. Am J Physiol 246: R161–183. 6696142 10.1152/ajpregu.1984.246.2.R161

[2] Borbely AA (2001) From slow waves to sleep homeostasis: new perspectives. Arch Ital Biol 139: 53–61. 11256187

[3] Brown RE, Basheer R, McKenna JT, Strecker RE, McCarley RW (2012) Control of sleep and wakefulness. Physiol Rev 92: 1087–1187. 22811426 10.1152/physrev.00032.2011 PMC3621793

[4] Tononi G, Cirelli C (2006) Sleep function and synaptic homeostasis. Sleep Med Rev 10: 49–62. 16376591 10.1016/j.smrv.2005.05.002

[5] Tononi G (2009) Slow wave homeostasis and synaptic plasticity. J Clin Sleep Med 5: S16–19. 19998870 PMC2824212

[6] Bushey D, Tononi G, Cirelli C (2011) Sleep and synaptic homeostasis: structural evidence in Drosophila. Science 332: 1576–1581. 21700878 10.1126/science.1202839 PMC3128387

[7] Huang ZL, Urade Y, Hayaishi O (2007) Prostaglandins and adenosine in the regulation of sleep and wakefulness. Curr Opin Pharmacol 7: 33–38. 17129762 10.1016/j.coph.2006.09.004

[8] Bjorness TE, Greene RW (2009) Adenosine and sleep. Curr Neuropharmacol 7: 238–245. 20190965 10.2174/157015909789152182 PMC2769007

[9] Porkka-Heiskanen T, Kalinchuk AV (2011) Adenosine, energy metabolism and sleep homeostasis. Sleep Med Rev 15: 123–135. 20970361 10.1016/j.smrv.2010.06.005

[10] Chikahisa S, Tominaga K, Kawai T, Kitaoka K, Oishi K, et al (2008) Bezafibrate, a peroxisome proliferator-activated receptors agonist, decreases body temperature and enhances electroencephalogram delta-oscillation during sleep in mice. Endocrinology 149: 5262–5271. 18787029 10.1210/en.2008-0285

[11] Chikahisa S, Fujiki N, Kitaoka K, Shimizu N, Sei H (2009) Central AMPK contributes to sleep homeostasis in mice. Neuropharmacology 57: 369–374. 19615388 10.1016/j.neuropharm.2009.07.015

[12] Bensinger SJ, Tontonoz P (2008) Integration of metabolism and inflammation by lipid-activated nuclear receptors. Nature 454: 470–477. 18650918 10.1038/nature07202

[13] Varga T, Czimmerer Z, Nagy L (2011) PPARs are a unique set of fatty acid regulated transcription factors controlling both lipid metabolism and inflammation. Biochim Biophys Acta 1812: 1007–1022. 21382489 10.1016/j.bbadis.2011.02.014 PMC3117990

[14] Poulsen L, Siersbaek M, Mandrup S (2012) PPARs: fatty acid sensors controlling metabolism. Semin Cell Dev Biol 23: 631–639. 22273692 10.1016/j.semcdb.2012.01.003

[15] Andersson U, Filipsson K, Abbott CR, Woods A, Smith K, et al (2004) AMP-activated protein kinase plays a role in the control of food intake. J Biol Chem 279: 12005–12008. 14742438 10.1074/jbc.C300557200

[16] Minokoshi Y, Shiuchi T, Lee S, Suzuki A, Okamoto S (2008) Role of hypothalamic AMP-kinase in food intake regulation. Nutrition 24: 786–790. 18725075 10.1016/j.nut.2008.06.002

[17] Hardie DG, Ross FA, Hawley SA (2012) AMPK: a nutrient and energy sensor that maintains energy homeostasis. Nat Rev Mol Cell Biol 13: 251–262. 22436748 10.1038/nrm3311 PMC5726489

[18] Wigren HK, Porkka-Heiskanen T (2009) Inducible nitric oxide synthase and AMP-activated protein kinase in basal forebrain during prolonged waking. Neuroreport 20: 97–101. 19033879 10.1097/WNR.0b013e32831af03d

[19] Dworak M, McCarley RW, Kim T, Kalinchuk AV, Basheer R (2010) Sleep and brain energy levels: ATP changes during sleep. J Neurosci 30: 9007–9016. 20592221 10.1523/JNEUROSCI.1423-10.2010 PMC2917728

[20] Godfrey KM, Barker DJ (2000) Fetal nutrition and adult disease. Am J Clin Nutr 71: 1344S–1352S. 10799412 10.1093/ajcn/71.5.1344s

[21] Gluckman PD, Hanson MA (2004) Living with the past: evolution, development, and patterns of disease. Science 305: 1733–1736. 15375258 10.1126/science.1095292

[22] Alamy M, Bengelloun WA (2012) Malnutrition and brain development: an analysis of the effects of inadequate diet during different stages of life in rat. Neurosci Biobehav Rev 36: 1463–1480. 22487135 10.1016/j.neubiorev.2012.03.009

[23] Kersten S, Seydoux J, Peters JM, Gonzalez FJ, Desvergne B, et al (1999) Peroxisome proliferator-activated receptor alpha mediates the adaptive response to fasting. J Clin Invest 103: 1489–1498. 10359558 10.1172/JCI6223 PMC408372

[24] Leone TC, Weinheimer CJ, Kelly DP (1999) A critical role for the peroxisome proliferator-activated receptor alpha (PPARalpha) in the cellular fasting response: the PPARalpha-null mouse as a model of fatty acid oxidation disorders. Proc Natl Acad Sci U S A 96: 7473–7478. 10377439 10.1073/pnas.96.13.7473 PMC22110

[25] Minokoshi Y, Alquier T, Furukawa N, Kim YB, Lee A, et al (2004) AMP-kinase regulates food intake by responding to hormonal and nutrient signals in the hypothalamus. Nature 428: 569–574. 15058305 10.1038/nature02440

[26] Maret S, Franken P, Dauvilliers Y, Ghyselinck NB, Chambon P, et al (2005) Retinoic acid signaling affects cortical synchrony during sleep. Science 310: 111–113. 16210540 10.1126/science.1117623

[27] Huang ZL, Qu WM, Eguchi N, Chen JF, Schwarzschild MA, et al (2005) Adenosine A2A, but not A1, receptors mediate the arousal effect of caffeine. Nat Neurosci 8: 858–859. 15965471 10.1038/nn1491

[28] Rodriguez JC, Gil-Gomez G, Hegardt FG, Haro D (1994) Peroxisome proliferator-activated receptor mediates induction of the mitochondrial 3-hydroxy-3-methylglutaryl-CoA synthase gene by fatty acids. J Biol Chem 269: 18767–18772. 7913466

[29] Hegardt FG (1999) Mitochondrial 3-hydroxy-3-methylglutaryl-CoA synthase: a control enzyme in ketogenesis. Biochem J 338 (Pt 3): 569–582. PMC1220089 10051425

[30] Kostiuk MA, Keller BO, Berthiaume LG (2010) Palmitoylation of ketogenic enzyme HMGCS2 enhances its interaction with PPARalpha and transcription at the Hmgcs2 PPRE. FASEB J 24: 1914–1924. 20124434 10.1096/fj.09-149765 PMC2874477

[31] Chakravarthy MV, Zhu Y, Lopez M, Yin L, Wozniak DF, et al (2007) Brain fatty acid synthase activates PPARalpha to maintain energy homeostasis. J Clin Invest 117: 2539–2552. 17694178 10.1172/JCI31183 PMC1937501

[32] Saper CB, Scammell TE, Lu J (2005) Hypothalamic regulation of sleep and circadian rhythms. Nature 437: 1257–1263. 16251950 10.1038/nature04284

[33] Stenberg D (2007) Neuroanatomy and neurochemistry of sleep. Cell Mol Life Sci 64: 1187–1204. 17364141 10.1007/s00018-007-6530-3 PMC11136155

[34] Jimenez-Chillaron JC, Hernandez-Valencia M, Lightner A, Faucette RR, Reamer C, et al (2006) Reductions in caloric intake and early postnatal growth prevent glucose intolerance and obesity associated with low birthweight. Diabetologia 49: 1974–1984. 16761107 10.1007/s00125-006-0311-7

[35] Isganaitis E, Jimenez-Chillaron J, Woo M, Chow A, DeCoste J, et al (2009) Accelerated postnatal growth increases lipogenic gene expression and adipocyte size in low-birth weight mice. Diabetes 58: 1192–1200. 19208909 10.2337/db08-1266 PMC2671035

