# Supplementary material for: Maternal Dietary Restriction Alters Offspring’s Sleep Homeostasis
Source: PLoS One. 2013 May 31;8(5):e64263. doi: 10.1371/journal.pone.0064263 (PMC3669365; doi:10.1371/journal.pone.0064263)
Supplement: Figure S1 — The influence of dietary restriction during gestation on maternal body weight changes, blood glucose, and live birth. Body weight changes before and after parturition in mother mice (A). Maternal blood glucose concentration (B) on gestation day 17. Live births (C), dead births (D), and ratio of male to female live births (E). Open bars and circles indicate AD mice. Closed bars and circles indicate DR mice. Data represent means ± SEM (A; n = 6–9, B; n = 2, C, D; n = 11, E; n = 7–8). **p<0.01 and *p<0.05 indicate a significant difference. (PPTX) [file pone.0064263.s001.pptx]

## Slide 1
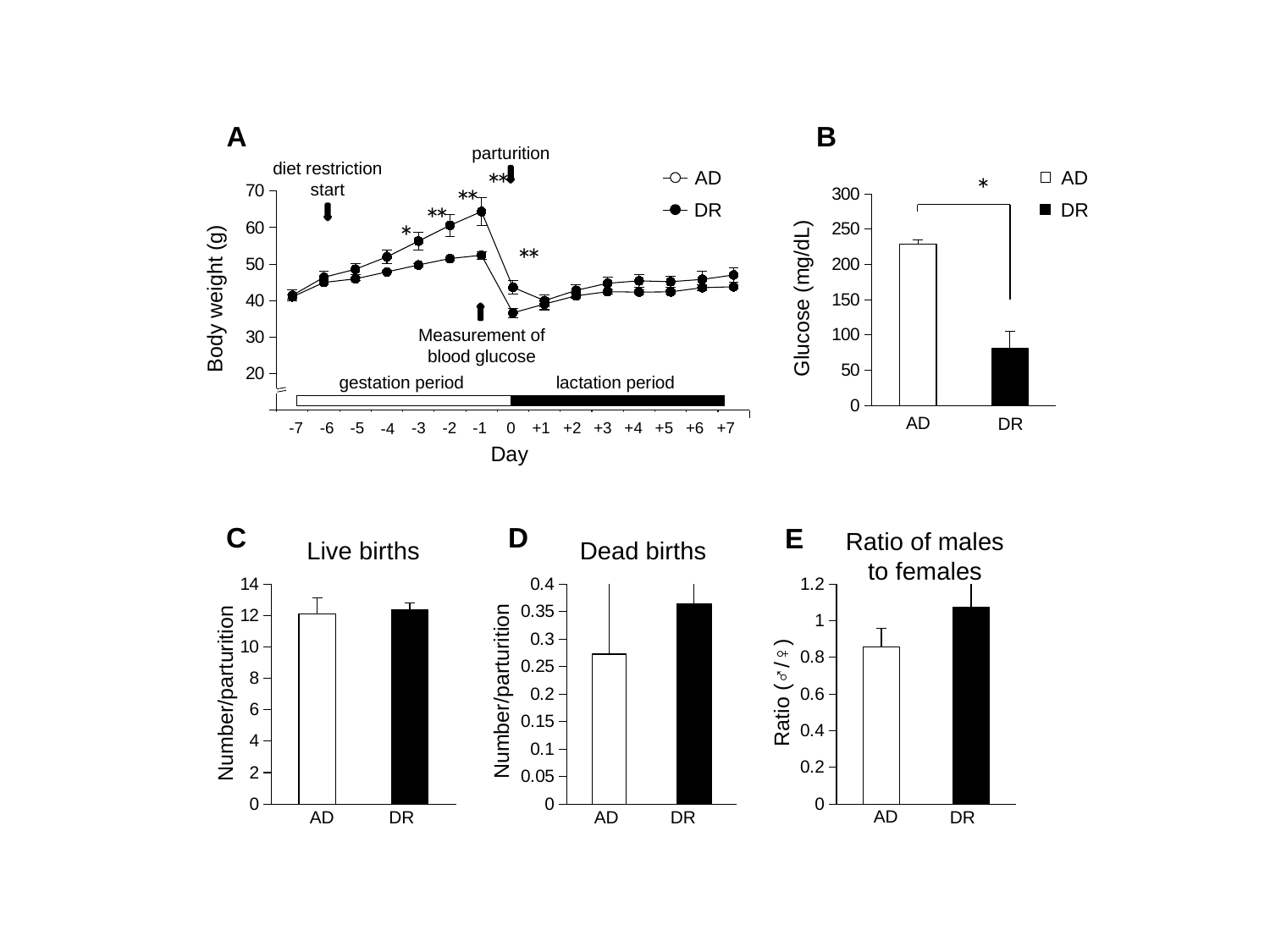

A
B
### Chart
| Category | |
|---|---|
| 0 | 229.0 |
| 0.5 | 80.5 |parturition
diet restriction start
　*
　*
AD
DR
AD
DR
　*
　*
　*
### Chart
| Category | | |
|---|---|---|　*
　*
　*
　*
　*
Body weight (g)
Glucose (mg/dL)
Measurement of blood glucose
lactation period
gestation period
0
AD
DR
-5
+1
+5
-7
-1
+4
-3
-6
+2
+3
+6
+7
-2
0
-4
Day
D
C
E
Ratio of males to females
Live births
Dead births
### Chart
| Category | |
|---|---|
| AD | 12.0909090909091 |
| DR | 12.36363636363637 |
### Chart
| Category | |
|---|---|
| AD | 0.272727272727273 |
| DR | 0.363636363636364 |
### Chart
| Category | |
|---|---|
| AD | 0.857993197278912 |
| DR | 1.074404761904762 |Number/parturition
Ratio (♂/♀)
Number/parturition
AD
AD
DR
DR
AD
DR
