# Supplementary material for: Maternal Dietary Restriction Alters Offspring’s Sleep Homeostasis
Source: PLoS One. 2013 May 31;8(5):e64263. doi: 10.1371/journal.pone.0064263 (PMC3669365; doi:10.1371/journal.pone.0064263)
Supplement: Figure S2 — The influence of dietary restriction during gestation on delta power in NREM sleep (A, B) in adult offspring mice. Open circles indicate AD mice. Closed circles indicate DR mice. Data represent means ± SEM (A, B; n = 6). (PPTX) [file pone.0064263.s002.pptx]

## Slide 1
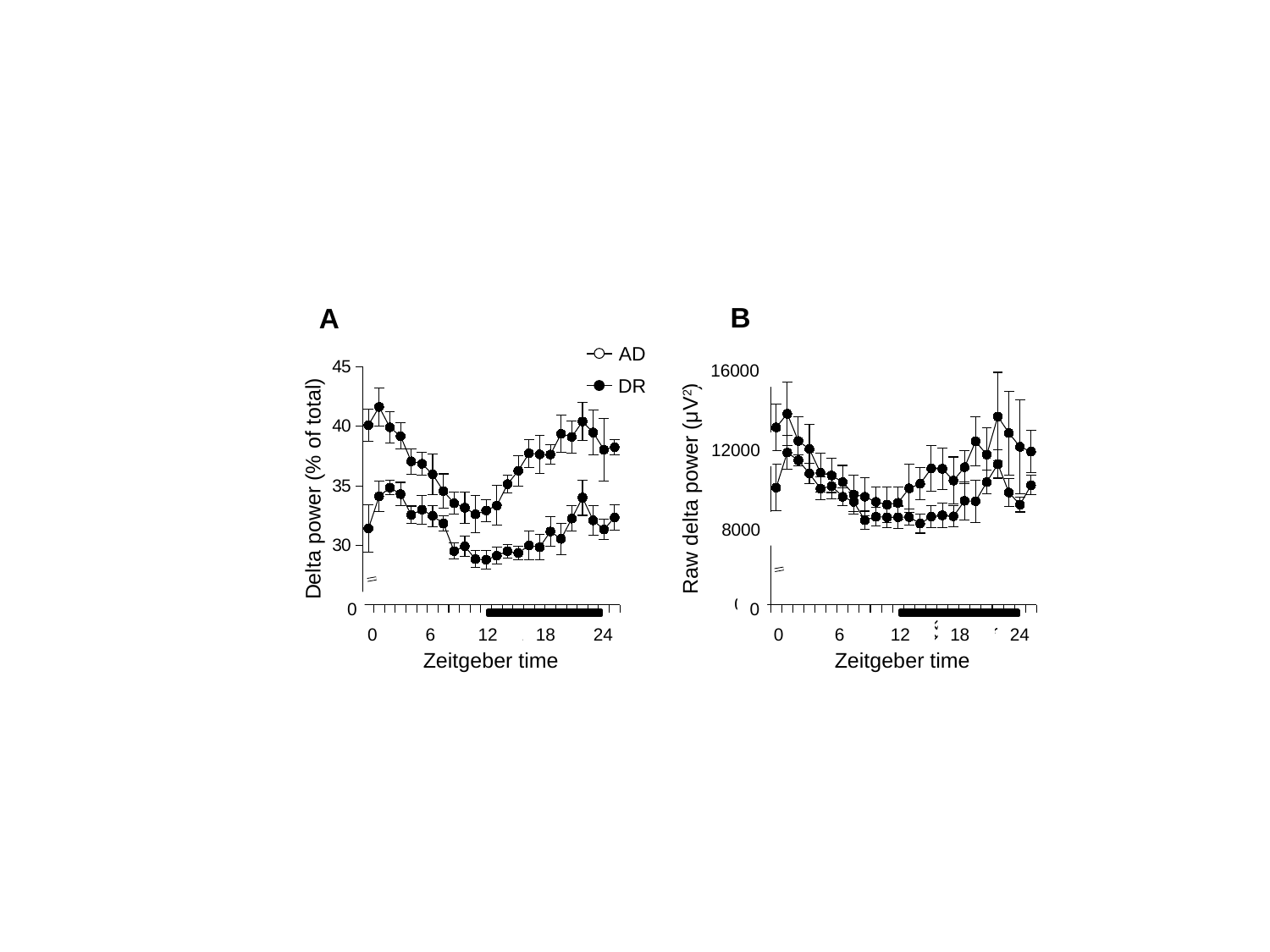

B
A
AD
DR
### Chart
| Category | CR (0%) | CR (50%) |
|---|---|---|
| 0 | 0.24747690295947 | 0.32330113740926 |
| 1 | 0.291649256131602 | 0.340451249526039 |
| 2 | 0.281875045951123 | 0.306217693415549 |
| 3 | 0.265193910294258 | 0.296033335024666 |
| 4 | 0.246383403909765 | 0.26615627932325 |
| 5 | 0.249472900355657 | 0.262783813625855 |
| 6 | 0.235982398606046 | 0.254438708386919 |
| 7 | 0.229541789575865 | 0.238750215646335 |
| 8 | 0.206366839299692 | 0.235942211352333 |
| 9 | 0.210877295442593 | 0.22953475525025 |
| 10 | 0.210123774765909 | 0.22599589044397 |
| 11 | 0.21016248716742 | 0.228426806556313 |
| 12 | 0.210427006378811 | 0.246679389007284 |
| 13 | 0.202187192266404 | 0.252358943613378 |
| 14 | 0.210887208580115 | 0.271742490762454 |
| 15 | 0.212580169301567 | 0.271286277710119 |
| 16 | 0.21134461026813 | 0.256423325333732 |
| 17 | 0.230823111809915 | 0.273262655503189 |
| 18 | 0.230310156846632 | 0.30572104623133 |
| 19 | 0.254411240276641 | 0.288949557176965 |
| 20 | 0.277129158329951 | 0.336971181430344 |
| 21 | 0.241351769750787 | 0.316246321656183 |
| 22 | 0.225993212145508 | 0.298879304409643 |
| 23 | 0.250606855707391 | 0.292838749818241 |
### Chart
| Category | CR (0%) | CR (50%) |
|---|---|---|
| 0 | 31.41986901239179 | 40.093933899193 |
| 1 | 34.11653723580594 | 41.61688308101226 |
| 2 | 34.84494526391964 | 39.90854418176573 |
| 3 | 34.30424366249724 | 39.1631127023345 |
| 4 | 32.55466419097834 | 37.05169416271588 |
| 5 | 32.99110734413898 | 36.84781360430618 |
| 6 | 32.47212626068423 | 35.9603295643125 |
| 7 | 31.83209093117198 | 34.55684813634852 |
| 8 | 29.52035627924597 | 33.53620268372183 |
| 9 | 29.91442209749309 | 33.15711471573996 |
| 10 | 28.84632077147391 | 32.60457485656732 |
| 11 | 28.79034199093908 | 32.9263728341926 |
| 12 | 29.1275248977932 | 33.34435806252933 |
| 13 | 29.50497044543386 | 35.13629505639403 |
| 14 | 29.34986964315954 | 36.2669850597438 |
| 15 | 29.99146899982129 | 37.72808991454334 |
| 16 | 29.84380232080025 | 37.63929498066177 |
| 17 | 31.15960346554298 | 37.62198408542675 |
| 18 | 30.5387256369288 | 39.36059605783596 |
| 19 | 32.2436752910144 | 39.10093283695356 |
| 20 | 34.00758730150275 | 40.39520699229801 |
| 21 | 32.09664144366396 | 39.46897065184508 |
| 22 | 31.32549014621348 | 38.03001173951426 |
| 23 | 32.33957190582874 | 38.24701093798438 |16000
12000
Raw delta power (μV2)
Delta power (% of total)
8000
0
0
12
18
24
0
6
9
11
12
6
8
7
10
12
18
24
0
6
Zeitgeber time
Zeitgeber time
