# Supplementary material for: Maternal Dietary Restriction Alters Offspring’s Sleep Homeostasis
Source: PLoS One. 2013 May 31;8(5):e64263. doi: 10.1371/journal.pone.0064263 (PMC3669365; doi:10.1371/journal.pone.0064263)
Supplement: Figure S3 — Threshold for waking by external stimuli (lights off) in adult offspring mice. The latency for awaking against lights-off conditions. Open bars indicate AD mice. Closed bars indicate DR mice. Data represent means ± SEM (n = 6). (PPTX) [file pone.0064263.s003.pptx]

## Slide 1
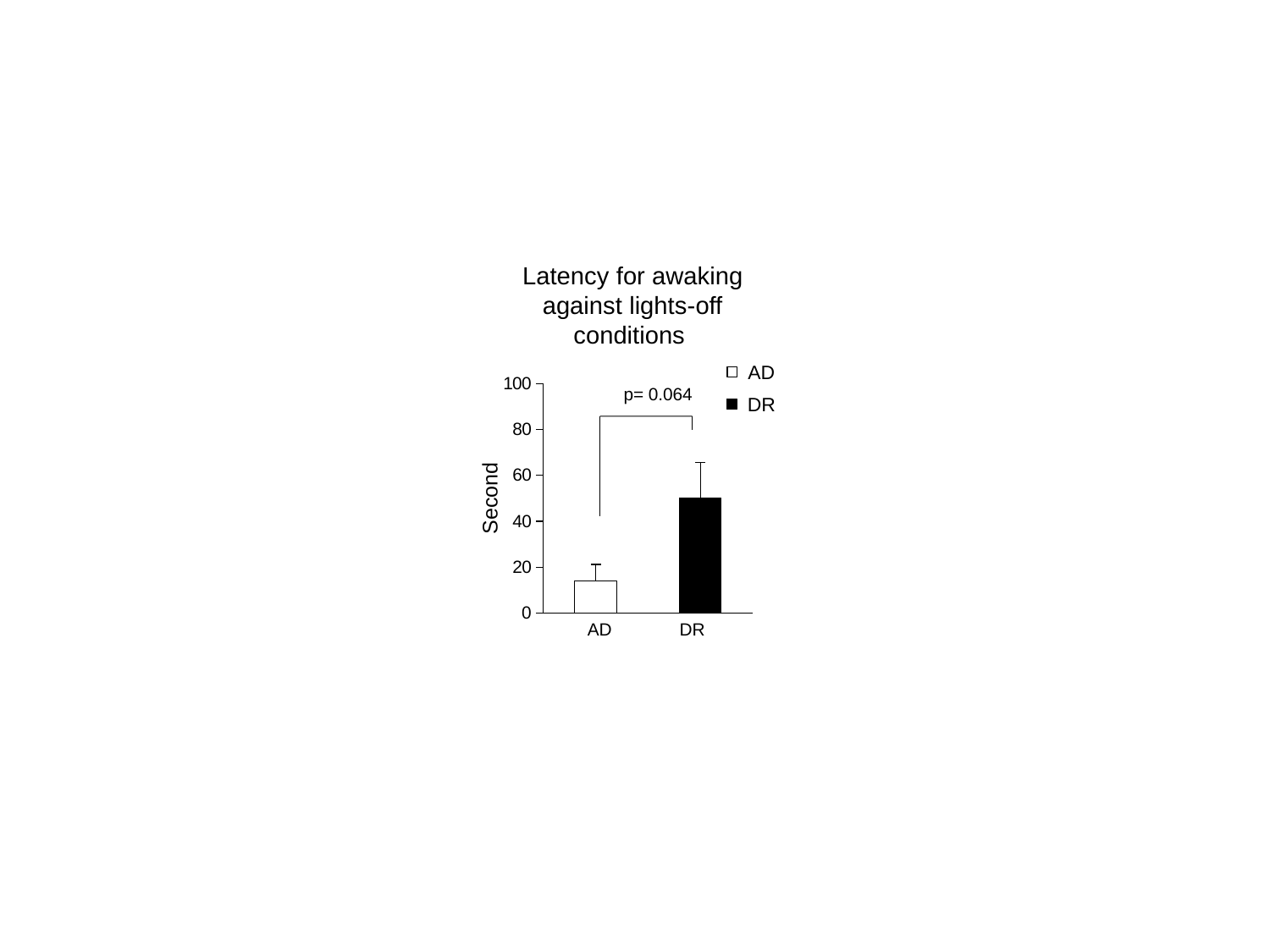

Latency for awaking against lights-off
conditions
AD
DR
### Chart
| Category | |
|---|---|
| AD | 14.0 |
| DR | 50.0 |　p= 0.064
Second
AD
DR
