# Supplementary material for: Maternal Dietary Restriction Alters Offspring’s Sleep Homeostasis
Source: PLoS One. 2013 May 31;8(5):e64263. doi: 10.1371/journal.pone.0064263 (PMC3669365; doi:10.1371/journal.pone.0064263)
Supplement: Figure S4 — The influence of dietary restriction during gestation on anxiety- and depression-like behaviors in adult offspring mice. Anxiety-like behavior was assessed by open field test, light-dark transition, and elevated plus maze. Time spent in the center area (A), total distance (B), and average speed (C) were assessed in the open field test. Number of transitions (D), latency to enter the light area for the first time (E), and time spent in the light area (F) were evaluated in the light-dark transition test. On the elevated-plus maze, time spent in open arms (G) and number of entries into open arms (H) were evaluated. Depression-like behavior was assessed by the forced swim test. Immobility time (I) was evaluated. Open bars indicate AD mice. Closed bars indicate DR mice. Data represent means ± SEM (A–I; n = 14). **p<0.01 and *p<0.05 indicate a significant difference. (PPTX) [file pone.0064263.s004.pptx]

## Slide 1
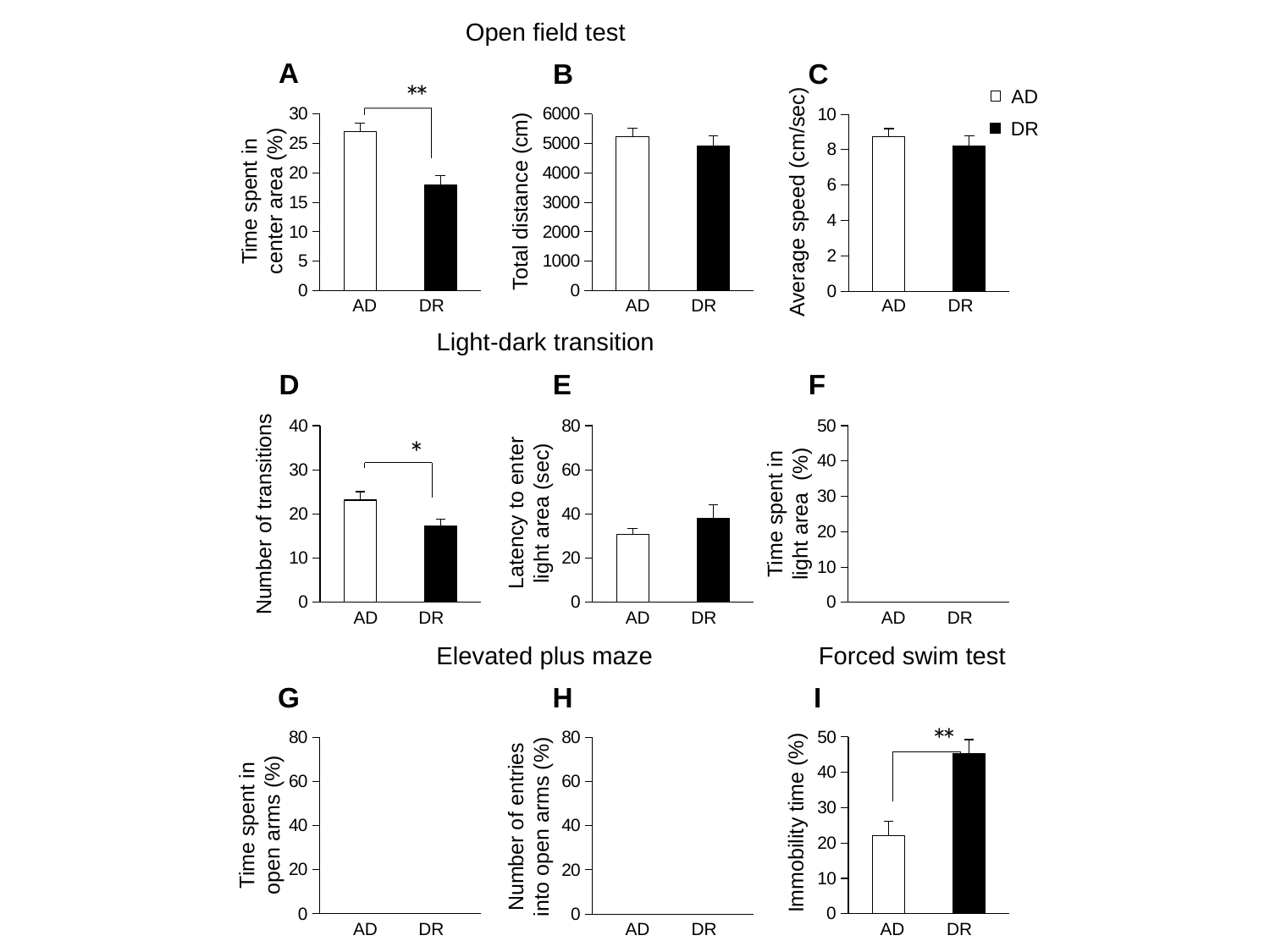

Open field test
A
C
B
　*
　*
AD
DR
### Chart
| Category | TotalDistance |
|---|---|
| 100%_WT | 5238.757142857144 |
| 50%_WT | 4914.957142857143 |
### Chart
| Category | TotalCenterTime(%) |
|---|---|
| 100%_WT | 26.97023809523809 |
| 50%_WT | 18.00595238095238 |
### Chart
| Category | AverageSpeed |
|---|---|
| 100%_WT | 8.735714285714286 |
| 50%_WT | 8.200000000000001 |Time spent in center area (%)
Total distance (cm)
Average speed (cm/sec)
AD
DR
AD
DR
AD
DR
Light-dark transition
D
E
F
### Chart
| Category | Light-Dark Test Number_of_Transitions |
|---|---|
| 100%_WT | 23.14285714285723 |
| 50%_WT | 17.28571428571405 |
### Chart
| Category | Light-Dark Test Latency_to_Transition |
|---|---|
| 100%_WT | 30.64285714285723 |
| 50%_WT | 38.07142857142829 |
### Chart
| Category | Light-Dark Test Light Time(%) |
|---|---|
| 100%_WT | 37.72166275899031 |
| 50%_WT | 34.78957122623315 |
　*
Latency to enter light area (sec)
Time spent in light area (%)
Number of transitions
AD
DR
AD
DR
AD
DR
Forced swim test
Elevated plus maze
I
H
G
　*
　*
### Chart
| Category | TotalFreezPercent(%) |
|---|---|
| 100%_WT | 22.12857142857143 |
| 50%_WT | 45.30714285714242 |
### Chart
| Category | Eleveited Pllus Maze (W+E)/Total(%) |
|---|---|
| 100%_WT | 34.51199559775317 |
| 50%_WT | 30.1735824551137 |
### Chart
| Category | Eleveited Pllus Maze (W+E)/Total(%)(NP) |
|---|---|
| 100%_WT | 30.1455144148074 |
| 50%_WT | 29.69902908051272 |Time spent in open arms (%)
Number of entries into open arms (%)
Immobility time (%)
AD
DR
AD
DR
AD
DR
