# Supplementary material for: Maternal Dietary Restriction Alters Offspring’s Sleep Homeostasis
Source: PLoS One. 2013 May 31;8(5):e64263. doi: 10.1371/journal.pone.0064263 (PMC3669365; doi:10.1371/journal.pone.0064263)
Supplement: Figure S5 — Monoaminergic system responsiveness in adult offspring mice. In vivo microdialysis. The change in extracellular concentration of serotonin (5-HT), its metabolite (5-HIAA), and norepinephrine (NE) before and after the forced swim test (A–C) in the hippocampus. The change in extracellular concentration of dopamine (DA) and its metabolites (DOPAC, HVA) before and after the forced swim test (E–G) in the striatum. Gene expression related to the regulation of serotonin signaling (D) such as 5-hydroxytryptamine receptor 1A (HTR1A, encoded by Htr1a), 5-hydroxytryptamine receptor 2C (HTR2C, encoded by Htr2c), solute carrier family 6, member 4 (SLC6A4, encoded by Slc6a4), tryptophan hydroxylase 1 (TPH1, encoded by Tph1), tryptophan hydroxylase 2 (TPH2, encoded by Tph2), and monoamine oxidase A (MAOA, encoded by Maoa) in the hippocampus. Gene expression related to the regulation of dopamine signaling (H) such as dopamine receptor D1A (DRD1A, encoded by Drd1a), dopamine receptor D2 (DRD2, encoded by Drd2), dopamine receptor D5 (DRD5, encoded by Drd5), solute carrier family 6, member 3 (SLC6A3, encoded by Slc6a3), tyrosine hydroxylase (TH, encoded by Th), and catechol-O-methyltransferase (COMT, encoded by Comt) in the striatum. Open bars indicate AD mice. Closed bars indicate DR mice. Data represent means ± SEM (A–C; n = 4, D; n = 6, E–G; n = 4, H; n = 7). (PPTX) [file pone.0064263.s005.pptx]

## Slide 1
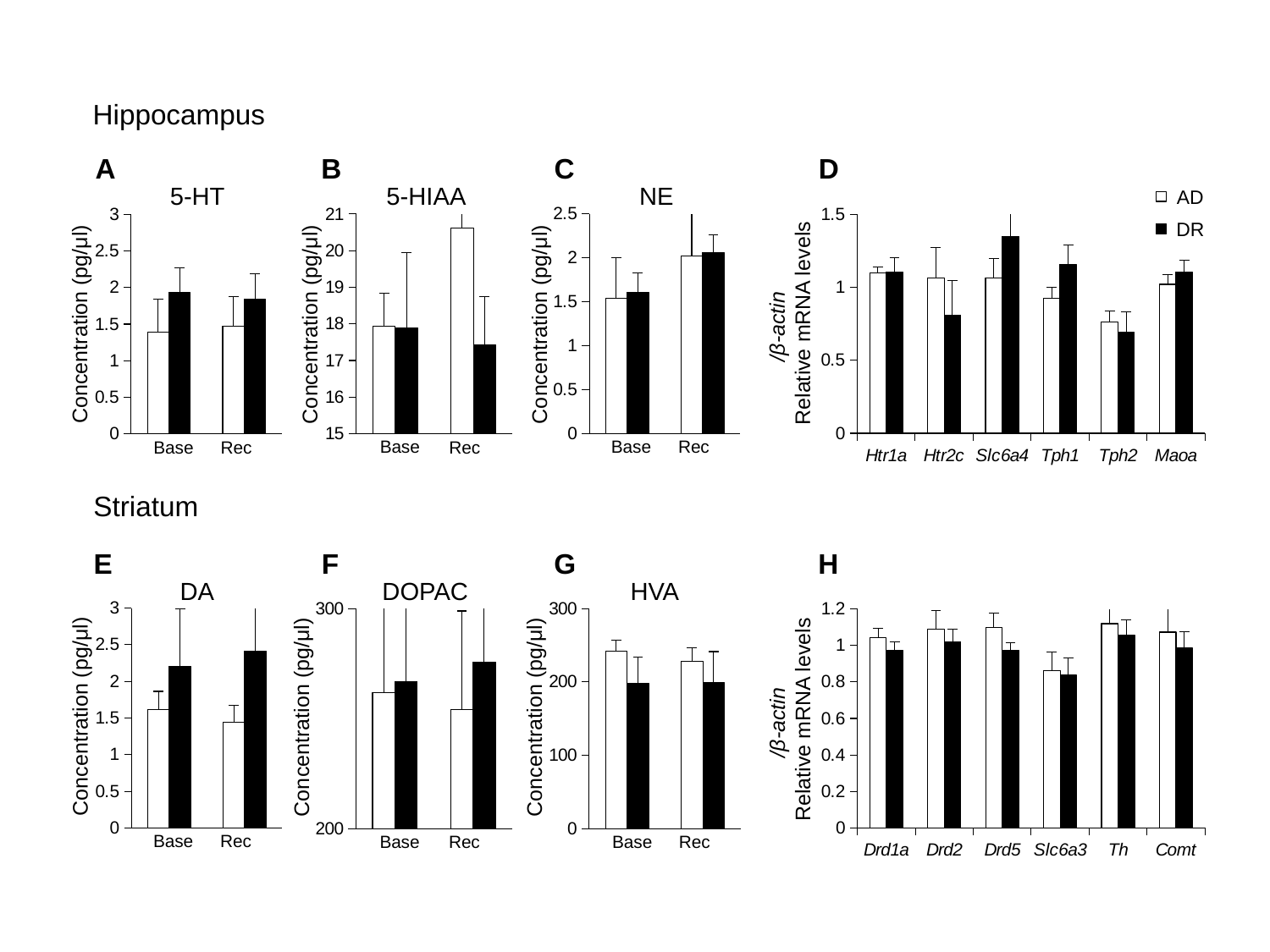

Hippocampus
B
C
D
A
5-HT
5-HIAA
NE
AD
DR
### Chart
| Category | 100% | 50% |
|---|---|---|
| Htr1a | 1.097634349803718 | 1.101817623300048 |
| Htr2c | 1.063983497989929 | 0.808266550341152 |
| Slc6a4 | 1.063061657571284 | 1.3485560493483 |
| Tph1 | 0.92572923544473 | 1.155380518693698 |
| Tph2 | 0.763058552442492 | 0.691061892949893 |
| Maoa | 1.019297269491365 | 1.104309903875912 |
### Chart
| Category | AD | 50%CR |
|---|---|---|
### Chart
| Category | AD | 50%CR |
|---|---|---|
### Chart
| Category | AD | 50%CR |
|---|---|---|Concentration (pg/μl)
Concentration (pg/μl)
Concentration (pg/μl)
/β-actin
 Relative mRNA levels
Base
Base
Rec
Rec
Base
Rec
Striatum
F
G
H
E
DA
DOPAC
HVA
### Chart
| Category | AD | 50%CR |
|---|---|---|
### Chart
| Category | 100% | 50% |
|---|---|---|
| Drd1a | 1.040819127565551 | 0.974603750218598 |
| Drd2 | 1.090029664012972 | 1.020530216052404 |
| Drd5 | 1.096865739797872 | 0.972838379714986 |
| Slc6a3 | 0.863475934952079 | 0.836235431936621 |
| Th | 1.118986314270415 | 1.053998760777235 |
| Comt | 1.072492472003834 | 0.984381615562763 |
### Chart
| Category | AD | 50%CR |
|---|---|---|
### Chart
| Category | AD | 50%CR |
|---|---|---|Concentration (pg/μl)
Concentration (pg/μl)
Concentration (pg/μl)
/β-actin
 Relative mRNA levels
Base
Rec
Base
Base
Rec
Rec
