# Supplementary figures and images for: Maternal Dietary Restriction Alters Offspring’s Sleep Homeostasis
Source: PLoS One. 2013 May 31;8(5):e64263. doi: 10.1371/journal.pone.0064263 (PMC3669365; doi:10.1371/journal.pone.0064263)

## Slide 1
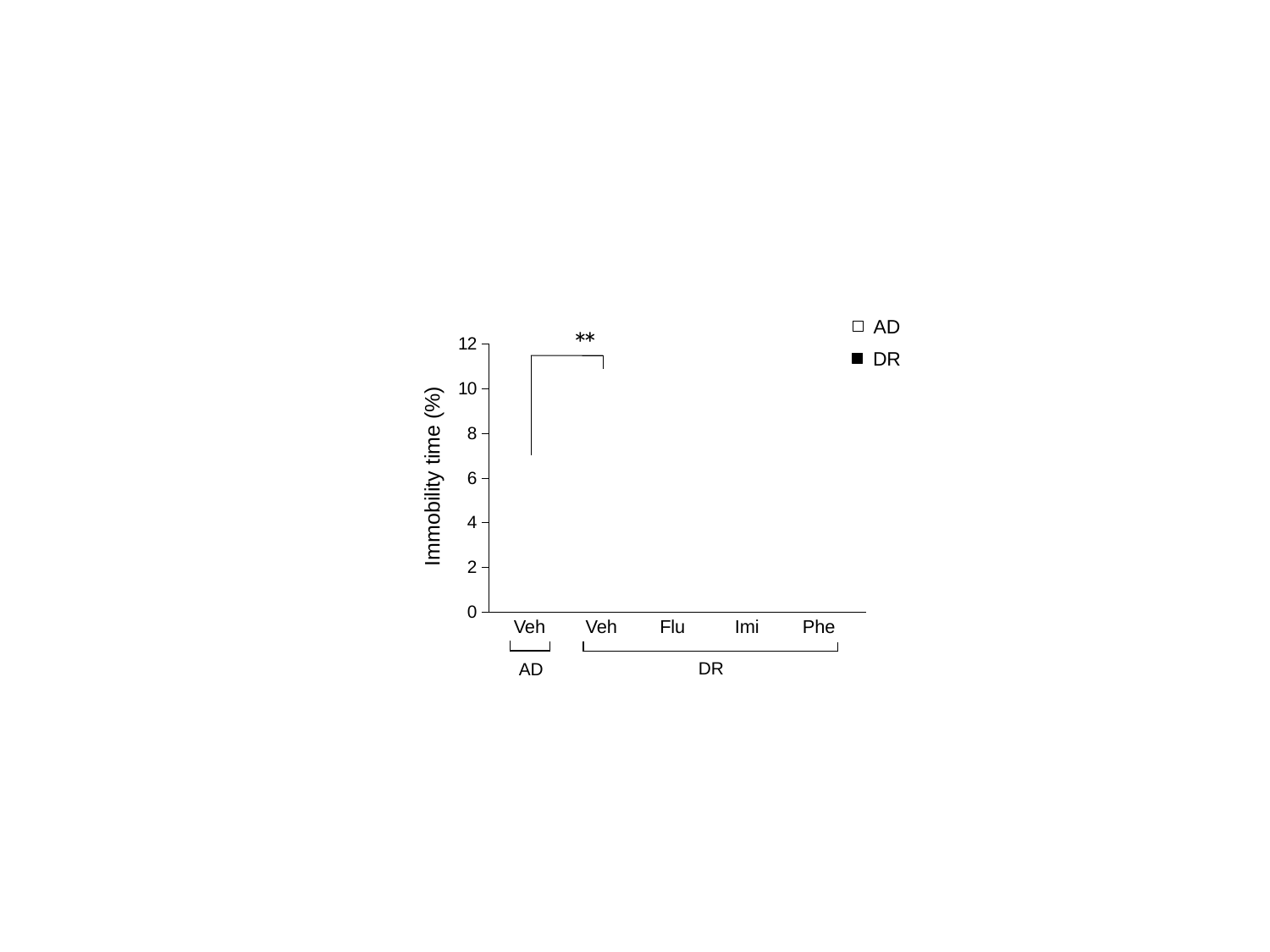

AD
DR
　*
　*
### Chart
| Category | |
|---|---|
| 100%_WT | 22.12857142857143 |
| 50%_WT | 45.30714285714281 |
| 50%_Fl | 31.03 |
| 50%_Imi | 33.36666666666654 |
| 50%_Phe | 44.86666666666654 |Immobility time (%)
Veh
Veh
Flu
Imi
Phe
DR
AD

Supplement: Figure S6 — Total time of immobility and changes in DR offspring mice treated with vehicle (Vhe; saline) or therapeutic drugs in the forced swim test. As therapeutic drugs, we used fluoxetine (Flu), imipramine (Imi), and phenelzine (Phe). Each therapeutic drug was injected intraperitoneally 30 min before the forced swim test. Open bars indicate AD mice. Closed bars indicate DR mice. Data represent means ± SEM (n = 9–10). **p<0.01 indicates a significant difference. (PPTX) [file pone.0064263.s006.pptx]

## Slide 1
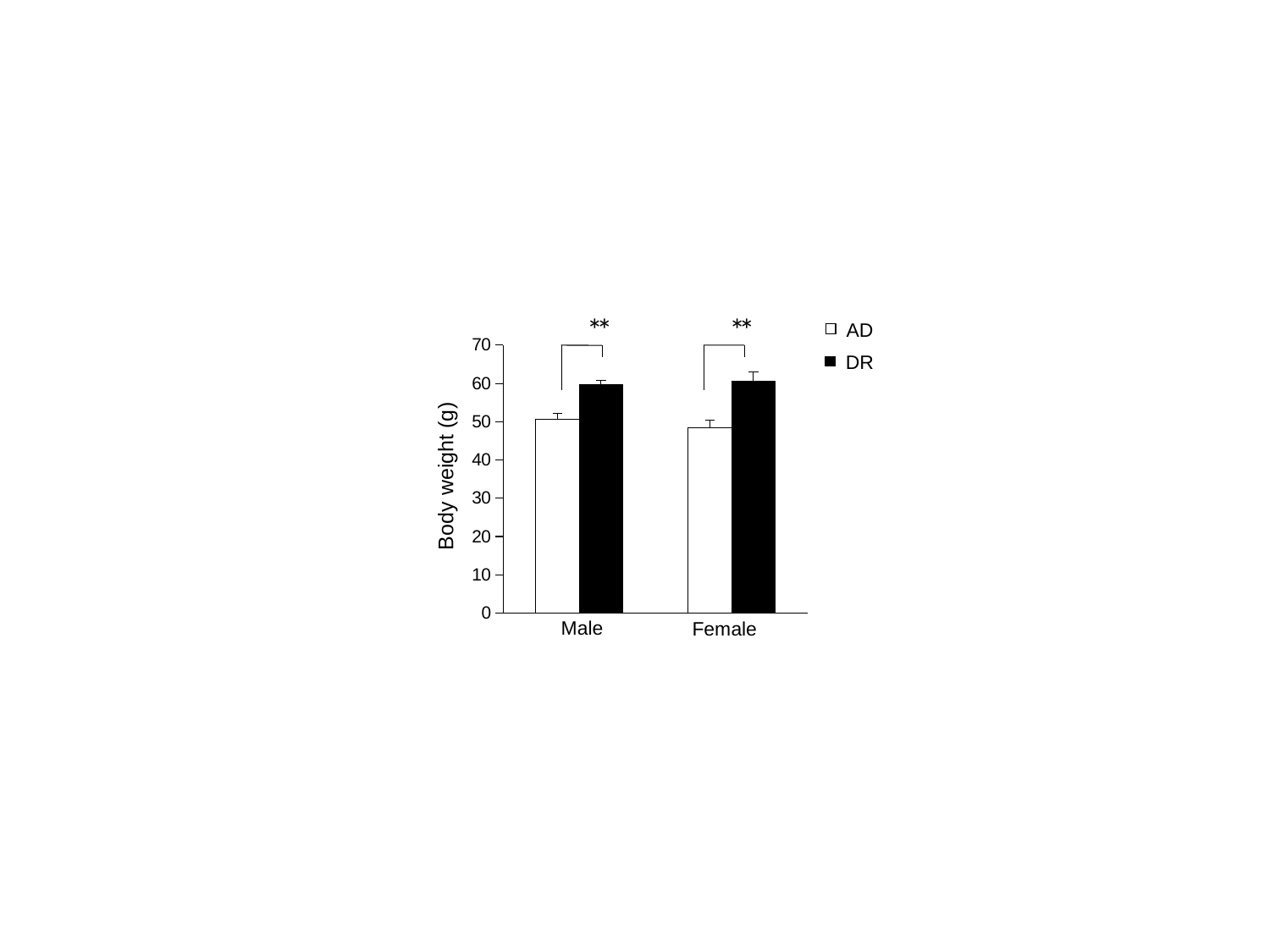

*
　*
　*
　*
AD
DR
### Chart
| Category | AD | DR |
|---|---|---|Body weight (g)
Male
Female

Supplement: Figure S10 — Body weight at adulthood in offspring male (aged 32 weeks) and female (aged 28–34 weeks) mice. Open bars indicate AD mice. Closed bars indicate DR mice. Data represent means ± SEM (n = 14–26). **p<0.01 indicates a significant difference. (PPTX) [file pone.0064263.s010.pptx]

## Slide 1
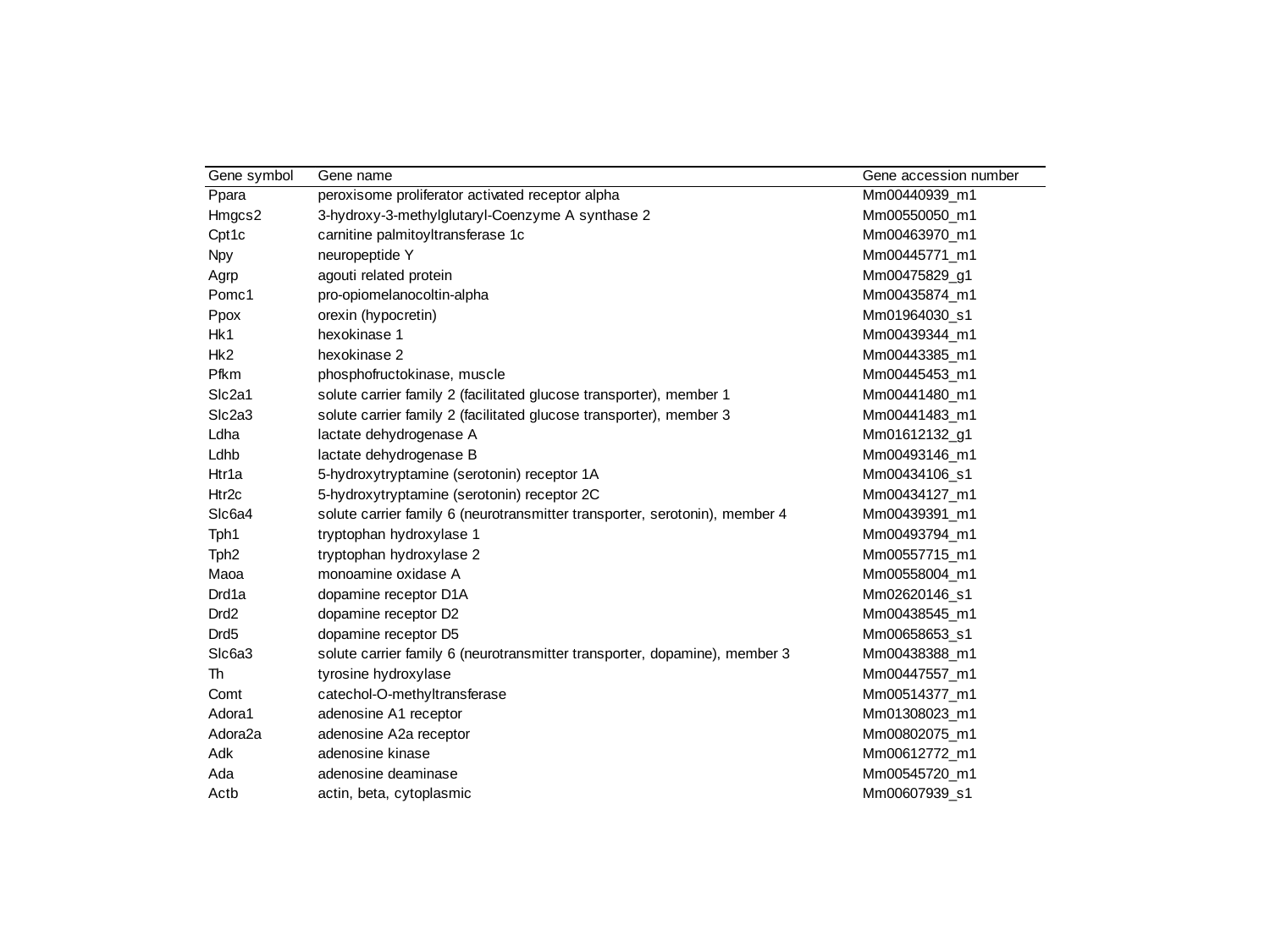

Supplement: Table S1 — List of gene-specific TaqMan probes and primers used for real-time RT-PCR. (PPTX) [file pone.0064263.s011.pptx]
