# Supplementary material for: Maternal Dietary Restriction Alters Offspring’s Sleep Homeostasis
Source: PLoS One. 2013 May 31;8(5):e64263. doi: 10.1371/journal.pone.0064263 (PMC3669365; doi:10.1371/journal.pone.0064263)
Supplement: Figure S7 — Gene expression related to the feeding regulation (A) and the regulation of glucose metabolism (B) such as hexokinase 1 (HK1, encoded by Hk1), hexokinase 2 (HK2, encoded by Hk2), phosphofructokinase, muscle (PFKM, encoded by Pfkm), solute carrier family 2, member 1 (SLC2A1, encoded by Slc2a1), solute carrier family 2, member 3 (SLC2A3, encoded by Slc2a3), lactate dehydrogenase A (LDHA, encoded by Ldha), and lactate dehydrogenase B (LDHB, encoded by Ldhb) in the hypothalamus. Open bars indicate AD mice. Closed bars indicate DR mice. Data represent means ± SEM (A, B; n = 6–7). (PPTX) [file pone.0064263.s007.pptx]

## Slide 1
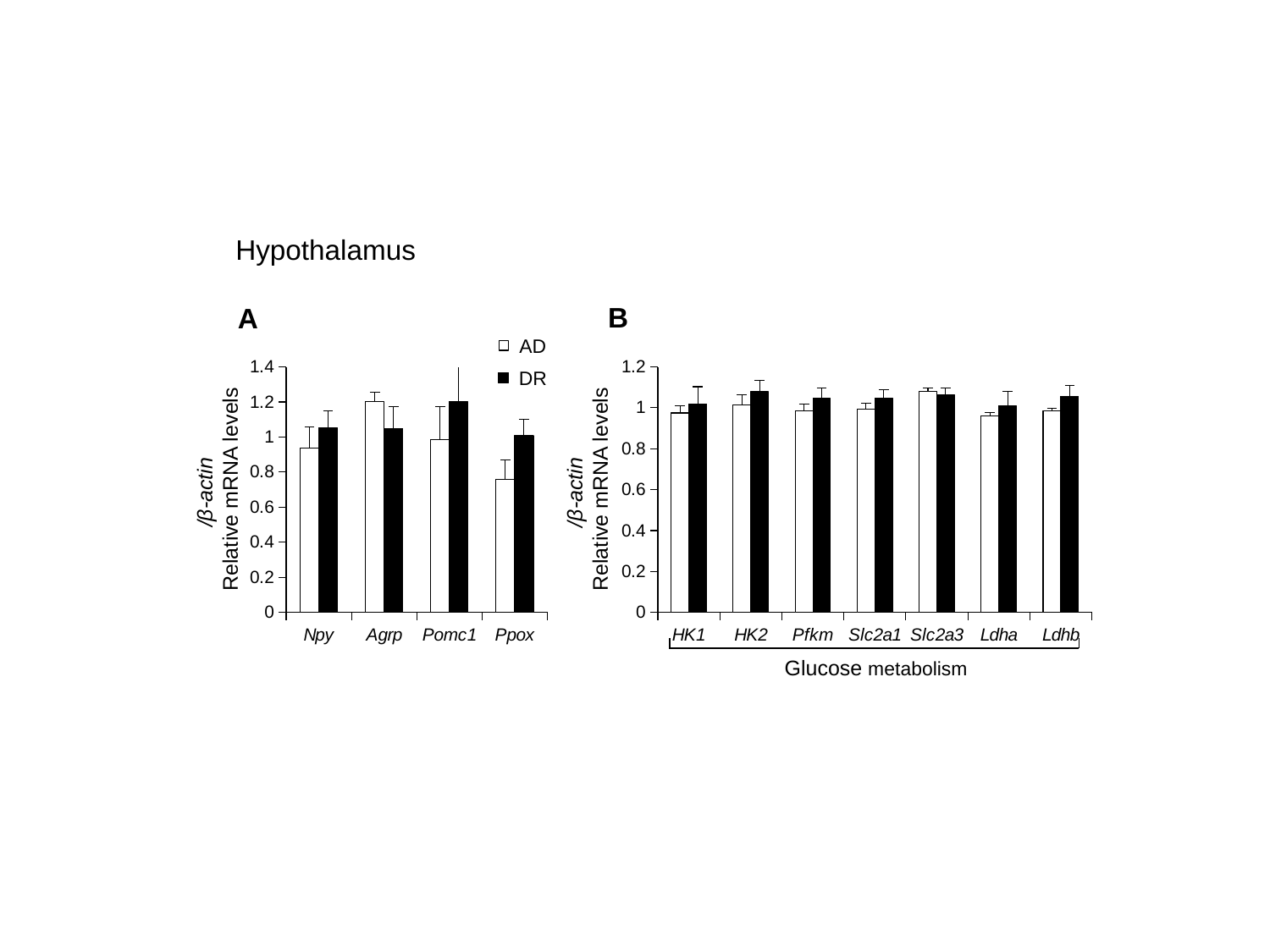

Hypothalamus
B
A
AD
DR
### Chart
| Category | 100% | 50% |
|---|---|---|
| Npy | 0.938531601287108 | 1.052031067539596 |
| Agrp | 1.203895076248574 | 1.048498621615383 |
| Pomc1 | 0.984455386308694 | 1.200983583365768 |
| Ppox | 0.759868395340755 | 1.006769185791639 |
### Chart
| Category | 100% | 50% |
|---|---|---|
| HK1 | 0.974923510882361 | 1.019539479789789 |
| HK2 | 1.015771330638405 | 1.078438132355836 |
| Pfkm | 0.985077137317274 | 1.047150140540984 |
| Slc2a1 | 0.99175953657058 | 1.048228430037325 |
| Slc2a3 | 1.079158304726067 | 1.062636585327692 |
| Ldha | 0.958730423144884 | 1.009350646849777 |
| Ldhb | 0.985437030527658 | 1.05706533500176 |/β-actin
 Relative mRNA levels
/β-actin
 Relative mRNA levels
Glucose metabolism
