# Supplementary material for: Maternal Dietary Restriction Alters Offspring’s Sleep Homeostasis
Source: PLoS One. 2013 May 31;8(5):e64263. doi: 10.1371/journal.pone.0064263 (PMC3669365; doi:10.1371/journal.pone.0064263)
Supplement: Figure S8 — The protein levels of AMPKα, p-AMPKα, and p-AMPKα/AMPKα ratio in the hypothalamus (A–D) and cortex (E–H). Open bars indicate AD mice. Closed bars indicate DR mice. Data represent means ± SEM (A–H; n = 5). (PPTX) [file pone.0064263.s008.pptx]

## Slide 1
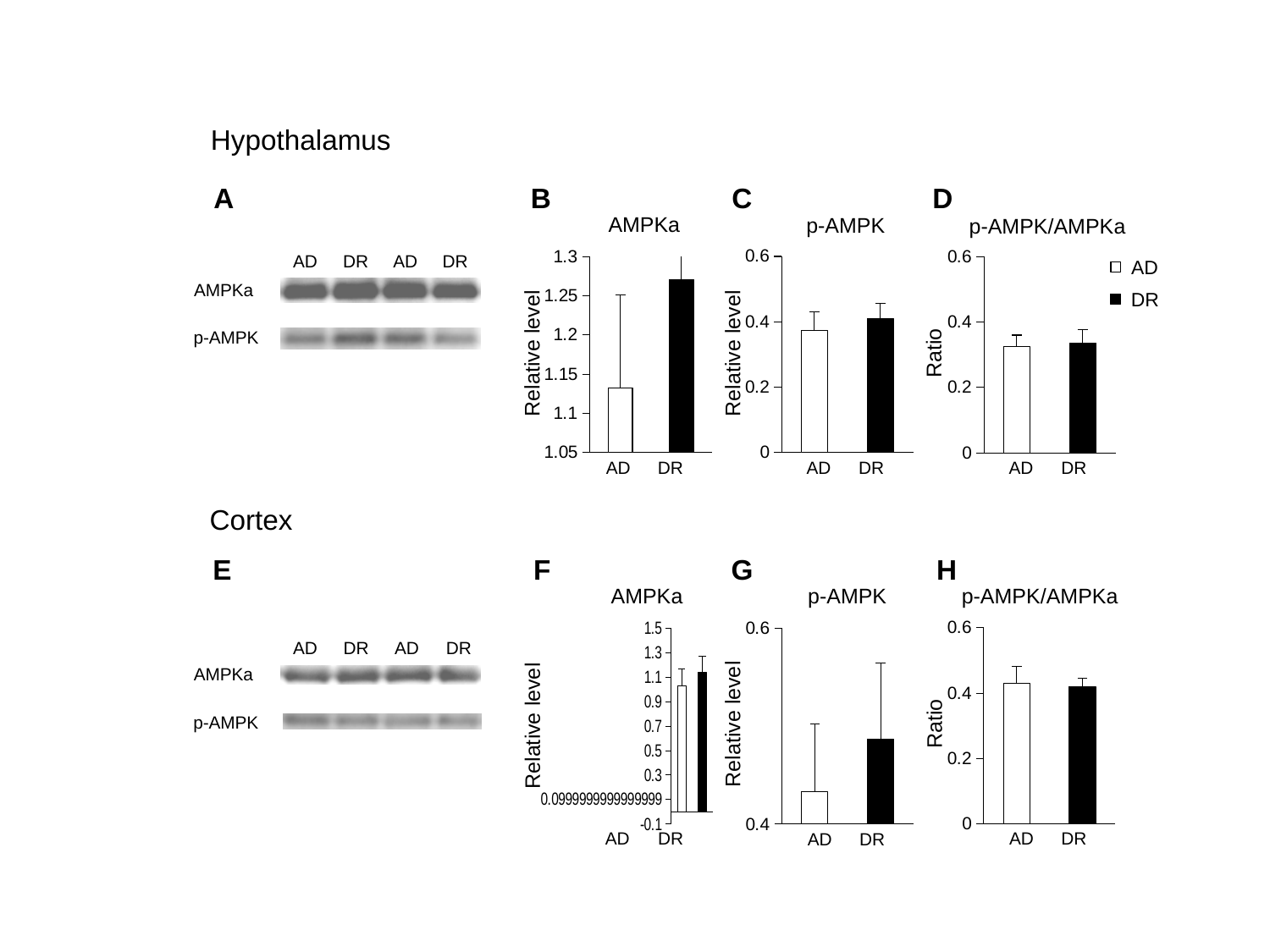

Hypothalamus
D
B
A
C
AMPKa
p-AMPK
p-AMPK/AMPKa
### Chart
| Category | |
|---|---|
| 1 | 0.372342400000002 |
| 0.5 | 0.4091684 |
### Chart
| Category | |
|---|---|
| 1 | 1.1322008 |
| 0.5 | 1.2707974 |
### Chart
| Category | |
|---|---|
| 1 | 0.32592946329705 |
| 0.5 | 0.335604542511775 |DR
AD
DR
AD
AD
DR
AMPKa
p-AMPK
Relative level
Relative level
Ratio
AD
DR
AD
DR
AD
DR
Cortex
F
H
G
E
p-AMPK/AMPKa
AMPKa
p-AMPK
### Chart
| Category | |
|---|---|
| 1 | 0.429283691316581 |
| 0.5 | 0.418460857125614 |
### Chart
| Category | |
|---|---|
| 1 | 1.027908199999993 |
| 0.5 | 1.138243 |
### Chart
| Category | |
|---|---|
| 1 | 0.433373200000002 |
| 0.5 | 0.4862662 |AD
DR
AD
DR
AMPKa
Relative level
Ratio
Relative level
p-AMPK
AD
DR
AD
DR
AD
DR
