# Supplementary material for: Maternal Dietary Restriction Alters Offspring’s Sleep Homeostasis
Source: PLoS One. 2013 May 31;8(5):e64263. doi: 10.1371/journal.pone.0064263 (PMC3669365; doi:10.1371/journal.pone.0064263)
Supplement: Figure S9 — The effect of 6-hour sleep deprivation on the mRNA expression of Pparα, Pparβ, and Pparγ in mouse brain. Open bars indicate control (Con) mice. Closed bars indicate sleep-deprived (SD) mice. Data represent means ± SEM (n = 6). *p<0.05 indicates a significant difference. (PPTX) [file pone.0064263.s009.pptx]

## Slide 1
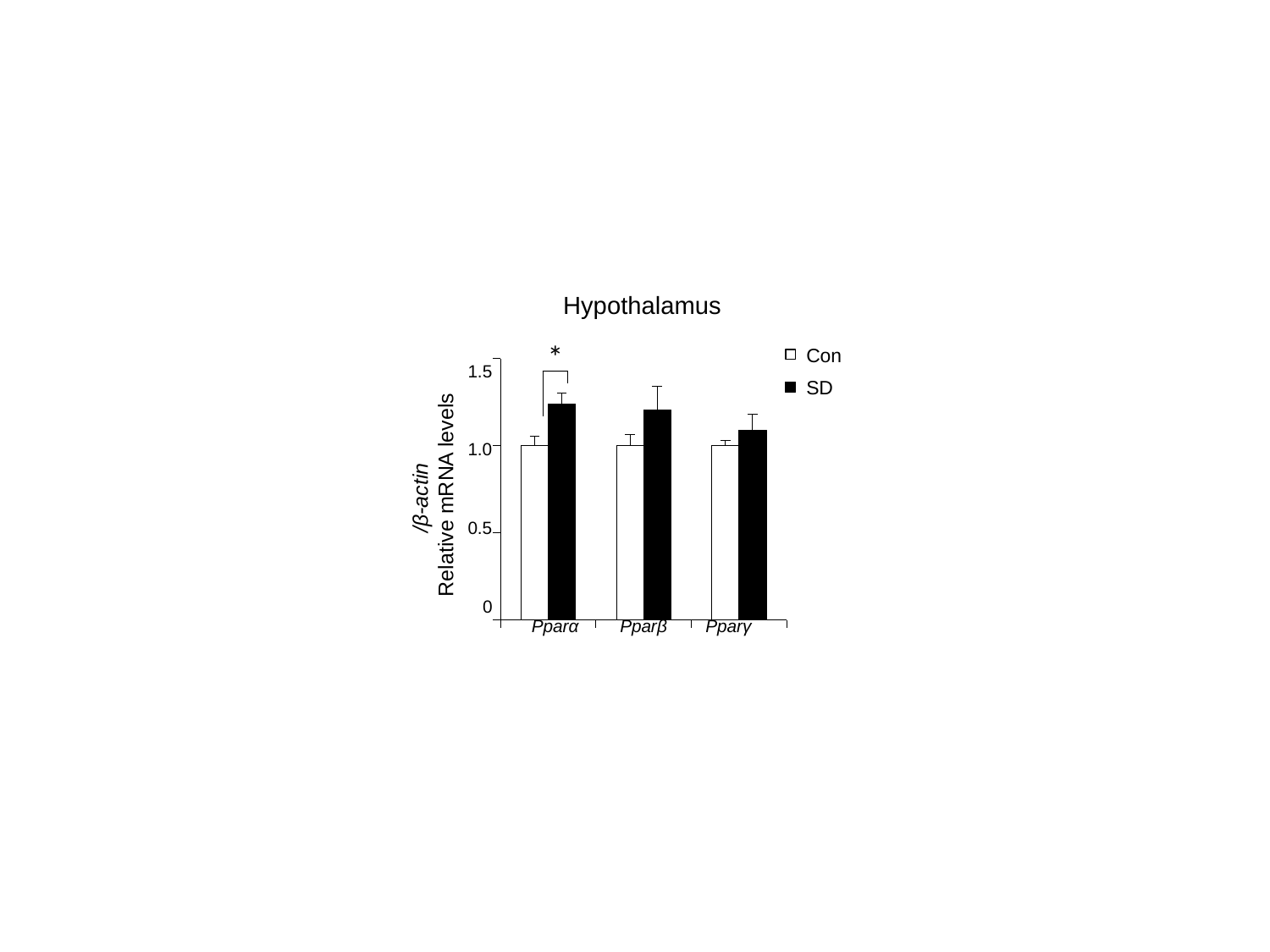

Hypothalamus
　*
### Chart
| Category | NS | SD |
|---|---|---|
| Ppara | 1.0 | 1.240015197375197 |
| Pparb | 1.0 | 1.204760495426833 |
| Pparg | 1.0 | 1.089421452880093 |1.5
1.0
/β-actin
 Relative mRNA levels
0.5
0
Pparα
Pparβ
Pparγ
Con
SD
