# Supplementary material for: Maternal Dietary Restriction Alters Offspring’s Sleep Homeostasis
Source: PLoS One. 2013 May 31;8(5):e64263. doi: 10.1371/journal.pone.0064263 (PMC3669365; doi:10.1371/journal.pone.0064263)
Supplement: Protocol S1 — Supplemental Methods. (DOC) [file pone.0064263.s012.doc]

**Supplemental Methods**

*Lights-off stimuli during sleep*

To estimate phenotypic sleep pressure in mice, we measured the waking threshold against not only cage shaking (Figure 4) but also lights-off conditions during sleep. Lights were turned off after the appearance of EEG delta wave activity. The latency from lights off to waking as determined by EEG and EMG was used as a parameter of sleep pressure.

*Behavioral tests*

To assess anxiety- and depression-like behavior, each behavioral test (open field test, light-dark transition, elevated plus maze, forced swim test) was carried out in AD and DR mice. Behavioral tests were performed between ZT4 and ZT8. Before each test, mice were habituated to the experimental room for at least 3 hours prior to the behavioral test. Mouse behaviors were videotaped and measured using an automated image analysis system, which was modified with the public domain Image J software. All experiments were done in a blinded and randomized fashion.

*Open field test*

Locomotor behavior was videotaped in an open field test chamber. The chamber was an open top box (45 cm × 45 cm × 30 cm) made of black acrylic, which was placed in the experimental room and illuminated by four indirect and homogenous lamps (150-200 lux). Each mouse was individually placed at a corner of the open field arena. Behavior in the chamber was monitored automatically for 10 min. The total distance and time spent in the central area were recorded automatically. Increased time spent in the central area has been shown to be an index of reduced anxiety-like behavior .

*Light-dark transition*

A chamber divided equally into two compartments (20 cm × 20 cm × 25 cm) was used. The light compartment (450-500 lux) consisted of a white floor and transparent walls and lid. The dark compartment consisted of a black floor, walls, and lid and was completely enclosed except for a small (5 cm × 3 cm) opening to allow movement between the dark and light compartment. Mice were placed in the dark compartment, and the latency to go out into the light compartment for the first time was recorded. The amount of time spent in each compartment was also recorded automatically for 5 min. A greater amount of time spent in the light compartment has been shown to be an index of reduced anxiety-like behavior .

*Elevated plus maze*

The plus maze consists of two connected runways. One of the runways contained the closed arms (5 cm × 25 cm) of the maze surrounded by walls 15 cm high. The other runway consisted of the open arms (5 cm × 25 cm) of the maze without walls. The maze was situated 135 cm above the floor. Each mouse was individually placed at the center of the maze facing the closed arm. Behavior of the mice in the maze was monitored for 10 min. The time spent and numbers of entries in the open arms were recorded automatically. Increased time spent and number of entries in the open arms has been shown to be an index of reduced anxiety-like behavior .

*Forced swim test*

Mice were individually placed in a plastic cylinder (6 cm × 22 cm) containing water 15 cm deep. Swimming behavior was monitored for 5 min. The duration of immobility was recorded automatically. When a mouse was observed floating in the water in an upright position without moving, it was considered to be immobile. Increased immobility time during the swim test has been shown to be an index of increased depression-like behavior .

*In vivo microdialysis*

Mice were anesthetized with a cocktail of ketamine (100 mg/kg) and xylazine (25 mg/kg). An intracerebral guide cannulae for the microdialysis probe was chronically implanted into the left ventral hippocampus (2.6 mm posterior to bregma, 3.5 mm lateral to the midline, -2.5 mm ventral to the skull surface) and/or the left ventral striatum (1.8 mm anterior to bregma, 0.6 mm lateral to the midline, -2.0 mm ventral to the skull surface) using a stereotaxic atlas . The detailed methods of measurement and data analysis were described previously . In the present study, microdialysis experiments were carried out in freely moving mice before, during, and after forced swim testing. The probe inlet cannula, connected to a microinfusion system (Eicom, Kyoto, Japan), was continuously perfused at a rate of 1μl/min with a Ringer solution. Dialysates were collected every 5 min from 25 min before to 25 min after the forced swim test. The mobile phase consisting of a mixture of 0.1 M citric acid and 0.1 M sodium acetate anhydrous (pH 3.9) containing 0.65 mM sodium 1-octanesulfonate, 13.4 μM EDTA Na2, and MeOH (83:17%, w/v) was used for dialysate analysis. The amounts of serotonin (5-hydroxytryptamine, 5-HT), 5-hydroxyindoleacetic acid (5-HIAA), norepinephrine (NE), dopamine (3, 4-dihydroxyphenethylamine, DA), 3, 4-dihydroxyphenylacetic acid (DOPAC), and homovanillic acid (HVA) were quantified via calibration curves derived from prepared standards.

*Pharmacological treatments and injection procedures*

In order to evaluate the effects of antidepressants in DR mice, fluoxetine (30 mg/kg, Sigma Chemical Co), imipramine (25 mg/kg, Sigma Chemical Co), and phenelzine (30 mg/kg, Sigma Chemical Co) were injected intraperitoneally 30 min before the forced swim test. Doses of each antidepressant were selected according to previous studies .

*Western blotting*

Cytoplasmic fractions of samples were collected using a Nuclear Extraction Kit (Cayman Chemical, Michigan, USA). Samples were fractionated by SDS-PAGE and transferred to a PVDF membrane. After blocking with 5% skimmed milk (Yukijirushi Nyugyo, Sapporo, Japan) dissolved in TBS-T (Tris-buffered saline with 0.1% Tween 20) for 1 h, the immobilized proteins were incubated overnight at 4 °C with primary antibodies. AMPKα anti-phospho-Thr172 (p-AMPKα) and anti-AMPKα (Cell Signaling Technology, Beverly, MA) were used at 1:1000 dilution. The anti-AMPKα reacts with both the α1 and α2 subunits. On the following day, the membranes were washed with TBS-T, and then incubated with horseradish peroxidase-conjugated secondary antibodies at 1:2000. After washing in TBS-T, blots were developed using an ECL kit (GE Healthcare, UK). Blots were scanned into a computer and analyzed using NIH Image 1.62 software. The band intensity for each sample was normalized to the mean value of all samples in each gel.

*Statistics*

Results are expressed as means ± SEM. Changes in body weight and EEG delta power were analyzed by repeated measures one-way or two-way analysis of variance (ANOVA) followed by Scheffe's post-hoc test. Results related to live births, dead births, male to female ratios, and latency for waking against lights-off conditions were analyzed by unpaired Student's t-test. The results for blood glucose measures, behavioral tests, microdialysis, real-time RT-PCR, and western blotting were analyzed by Mann-Whitney U test. P < 0.05 was assumed to indicate statistical significance.

**References**

1. Prut L, Belzung C (2003) The open field as a paradigm to measure the effects of drugs on anxiety-like behaviors: a review. Eur J Pharmacol 463: 3-33.

2. Bourin M, Hascoet M (2003) The mouse light/dark box test. Eur J Pharmacol 463: 55-65.

3. Lister RG (1987) The use of a plus-maze to measure anxiety in the mouse. Psychopharmacology (Berl) 92: 180-185.

4. Lucki I (1997) The forced swimming test as a model for core and component behavioral effects of antidepressant drugs. Behav Pharmacol 8: 523-532.

5. Franklin KBJ, Paxinos G (2008) The Mouse Brain in Stereotaxic Coodinates. San Diego, CA: Academic Press.

6. Sei H, Sano A, Oishi K, Fujihara H, Kobayashi H, et al. (2003) Increase of hippocampal acetylcholine release at the onset of dark phase is suppressed in a mutant mice model of evening-type individuals. Neuroscience 117: 785-789.

7. Griebel G, Curet O, Perrault G, Sanger DJ (1998) Behavioral effects of phenelzine in an experimental model for screening anxiolytic and anti-panic drugs: correlation with changes in monoamine-oxidase activity and monoamine levels. Neuropharmacology 37: 927-935.

8. Sugimoto Y, Furutani S, Nishimura K, Itoh A, Tanahashi T, et al. (2010) Antidepressant-like effects of neferine in the forced swimming test involve the serotonin1A (5-HT1A) receptor in mice. Eur J Pharmacol 634: 62-67.

9. Wang R, Xu Y, Wu HL, Li YB, Li YH, et al. (2008) The antidepressant effects of curcumin in the forced swimming test involve 5-HT1 and 5-HT2 receptors. Eur J Pharmacol 578: 43-50.
